# Dual‐Color Photoconvertible Fluorescent Probes Based on Directed Photooxidation Induced Conversion for Bioimaging

**DOI:** 10.1002/anie.202215085

**Published:** 2022-12-14

**Authors:** Lazare Saladin, Victor Breton, Ophélie Dal Pra, Andrey S. Klymchenko, Lydia Danglot, Pascal Didier, Mayeul Collot

**Affiliations:** ^1^ Laboratoire de Bioimagerie et Pathologies UMR 7021 CNRS/ Université de Strasbourg 74 route du Rhin 67401 Illkirch-Graffenstaden France; ^2^ Institute of Psychiatry and Neuroscience of Paris (IPNP) INSERM U1266 Membrane Traffic in Healthy and Diseased Brain Université Paris Cité 102 rue de la santé 75014 Paris France; ^3^ Institute of Psychiatry and Neuroscience of Paris (IPNP), INSERM U1266, Sientific director of NeurImag facility Université Paris Cité 102 rue de la santé 75014 Paris France

**Keywords:** Fluorescent Probes, Lipid Droplets, Photoconversion, Photooxidation, Photoswitching

## Abstract

We herein present a new concept to produce dual‐color photoconvertible probes based on a mechanism called Directed Photooxidation Induced Conversion (DPIC). As a support of this mechanism, styryl‐coumarins (**SCs**) bearing Aromatic Singlet Oxygen Reactive Moieties (ASORMs) like furan and pyrrole have been synthesized. **SCs** are bright fluorophores, which undergo a hypsochromic conversion upon visible light irradiation due to directed photooxidation of the ASORM that leads to the disruption of conjugation. **SC‐P**, a yellow emitting probe bearing a pyrrole moiety, converts to a stable blue emitting coumarin with a 68 nm shift allowing the photoconversion and tracking of lipid droplet in live cells. This new approach might pave the way to a new generation of photoconvertible dyes for advanced bioimaging applications.

## Graphical abstract

A method to obtain bright dual‐color photoconvertible fluorophores is presented based on a new mechanism called Directed Photooxidation Induced Conversion (DPIC). The mechanism is based on the directed oxidation of the pyrrole that is conjugated to the fluorophore. This approach led to SC‐P, which stains the lipid droplets in live cells and that undergoes a hypsochromic shift of 68 nm upon visible light irradiation.

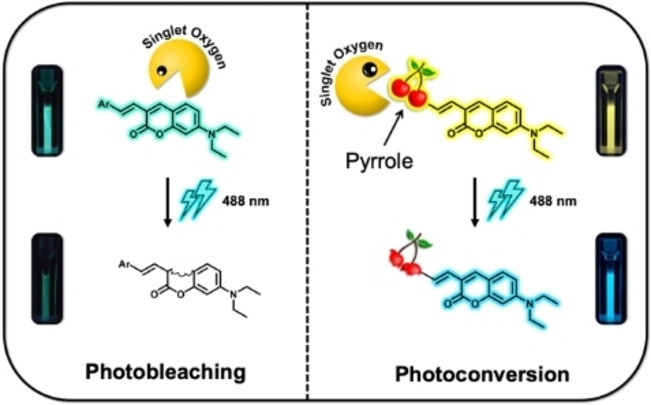

Photoconversion of fluorescent dyes in the visible range is a powerful tool in bioimaging to unambiguously track labeled biomolecules over large spatiotemporal scales. Although efficient photoconvertible fluorescent proteins have been developed,[^1^, ^2^] molecular probes, characterized by their small size, improved optical properties, versatility and ease of use constitute a complementary and robust tool in the field of bioimaging, as they provide a homogeneous staining in cells and can be used in tissue and in vivo imaging.[^3^, ^4^, ^5^] In this context, Dual‐Color Photoconvertible Fluorophores (DCPFs), able to convert from a bright emissive form to another, being advantageously detected prior to conversion, have recently drawn attention. Bright fluorophores like AlexaFluor 647^[6]^ and other cyanines^[7]^ have been evaluated as DCPFs by studying the emissive form resulting from partial photobleaching. Conversely to this empirical approach, DCPFs with rational designs have been proposed through various mechanisms including: dealkylation of rhodamines,^[8]^ phototruncation of cyanines,[^9^, ^10^, ^11^] photocyclodehydrogenation of diazaxanthilidene[^12^, ^13^] and AIEgen,^[14]^ spiropyranization of oxazine,^[15]^ and photooxidative dehydrogenation.^[16]^ Although these efforts led to efficient DCPFs, some limitations remain to be addressed including the undesired reversibility, low brightness of both initial and converted forms, irradiation with phototoxic UV, and low yields of conversion. Additionally, the kinetics of the photoconversion process should be adapted. Indeed, slow conversion could lead to phototoxicity of live samples, whereas fast conversion would make the probe unusable. Ideally, a more universal approach, applicable to various fluorophores, would enable to extend the palette of properties and available colors of DCPFs for various applications in bioimaging.

We thus aimed at establishing a rational design of DCPFs supported by a new mechanism. Herein, we introduce a strategy to obtain photoconvertible fluorophores based on Directed Photooxidation Induced Conversion (DPIC). We hypothesized that efficient DCPFs could be obtained from a directed partial photobleaching of the fluorophore leading to a hypsochromic shift of both excitation and emission spectra. Photobleaching occurs upon light irradiation where fluorophores generate singlet oxygen (^1^O_2_), which oxidizes the dye and mediate irreversible photochemical cleavage leading to non‐emissive species.^[17]^ We thus assumed that the conjugation of a fluorophore to an Aromatic Singlet Oxygen Reactive Moiety (ASORM) could 1) lead to a bathochromic shift by extension of the π system, and 2) upon generation of ^1^O_2_, direct the oxidation towards the ASORM, thus provoking a disruption of the conjugation, leading to a hypsochromic shift in excitation and emission (Figure 1).

**Figure 1:**
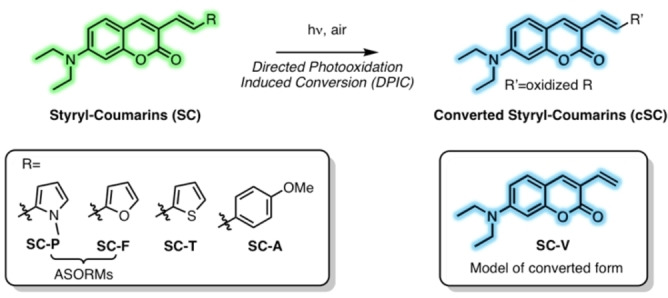
Principle of the Directed Photooxidation Induced Conversion (DPIC) where the conjugation between the coumarin and the Singlet Oxygen Reactive Moieties (ASORMs) is disrupted upon photooxidation of the latter. Structures of the Styryl‐Coumarins (**SC**), their converted forms after photoirradiation (**cSC**), and the model of converted form (**SC‐V**).

For our study, coumarin was chosen as small and bright blue emissive fluorophore. Coumarins display high extinction coefficients and fluorescence quantum yields with low cytotoxicity making them a suitable platform to develop fluorescent probes for bioimaging.^[18]^ Pyrrole^[19]^ and furan^[20]^ were chosen as ASORMs for their small size, and their ability to photooxidize in the presence of ^1^O_2_ resulting in dearomatization. The coumarin core fluorophore was thus conjugated to *N*‐methylpyrrole and furan through a Wittig reaction to obtain the extended styryl‐coumarins **SC‐P** and **SC‐F** respectively (Figure 1). As comparison, styryl coumarins thiophene (**SC‐T**) and anisole (**SC‐A**) were synthesized to demonstrate the unique effect of Singlet Oxygen Reactive Moiety (ASORM) in the photoconversion process (Figure 1).

Additionally, a vinyl coumarin (**SC‐V**) was synthesized as a model of product resulting from Directed Photooxidation Induced Conversion (Figure 1). The styryl coumarins were obtained in pure form and were characterized by ^1^H and ^13^C NMR as well as high resolution mass spectrometry.

The photophysical properties of the styryl coumarins (**SCs**) were evaluated in various solvents and showed high brightness ranging from 24 000 to 43 900 M^−1^ cm^−1^ with slight solvatochromic shifts (Figure S1, Table S1). In methanol, **SC‐T**, **SC‐F** and **SC‐A** displayed quite similar properties with λ_Abs_ and λ_Em_ around 425 and 512 nm respectively (Figure 2A), extinction coefficient (ϵ) around 30 000 M^−1^.cm^−1^ and fluorescence quantum yields up to 0.77 (Table 1), whereas **SC‐P** displayed a significant red‐shifted emission spectrum (Figure 2A, Table 1). As expected, **SC‐V** is a typical blue‐emissive coumarin (Figure 2A) though displaying a rather high brightness (Table 1: ϵ=25 000 M^−1^ cm^−1^, φ_f_=0.79).

**Figure 2:**
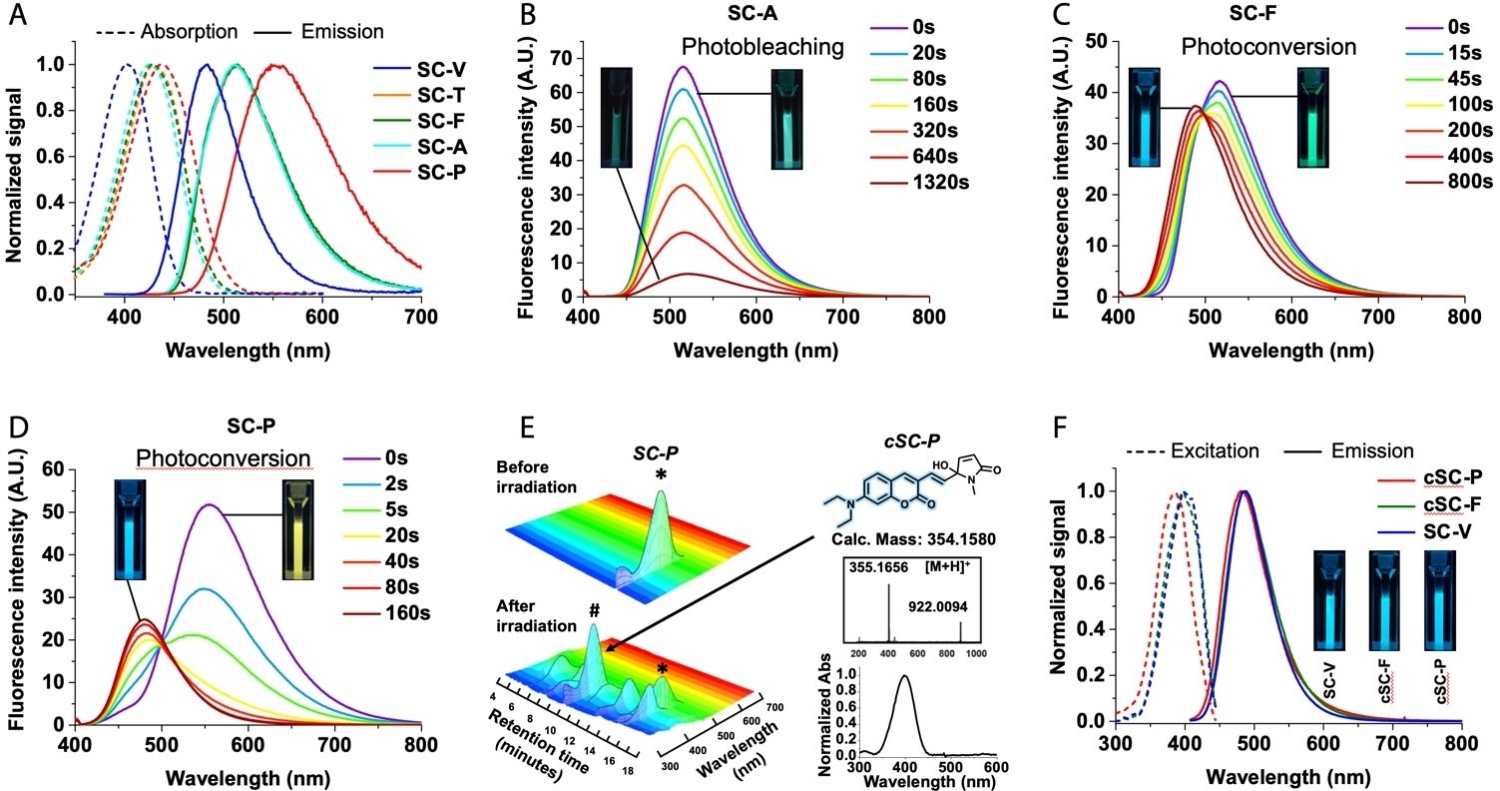
Spectroscopic studies and photoirradiation of **SCs** (5 μM in MeOH). (A) Absorption and emission spectra of **SCs**. (B–D) Emission spectra of **SCs** upon irradiation at 488 nm (power density: 33 mW cm^−2^). Whereas **SC‐A** undergoes photobleaching upon irradiation (B), **SC‐F** (C) and **SC‐P** (D) photoconvert towards new emissive species. (E) 3D HPLC‐UV/Visible‐mass characterization of **SC‐P** (star) before and after photoconversion. **cSC‐P** (Hashtag) is a proposed structure of one of the blue emitting photoproducts obtained after conversion based on the mass and absorption spectra below. (F) Normalized excitation (emission at 450 nm) and emission (excitation at 405 nm) spectra of converted forms **cSC‐F** and **cSC‐P** along with **SC‐V** showing similar photophysical properties. Insets are photographs under a UV lamp excitation of cuvette before and after irradiation.

**Table 1:**
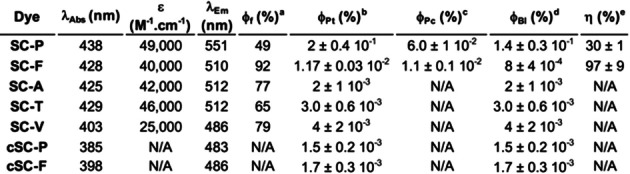
Photophysical properties of **SCs** and their photoproducts (**cSCs**).

[a] Fluorescence quantum yield. [b] Quantum yield of phototransformation. [c] Quantum yield of photoconversion. [d Quantum yield of photobleaching. [e] Chemical yield of photoconversion.

To evaluate their dual‐color conversion properties, methanolic solutions of dyes were irradiated with a continuous wave 488 nm‐laser and emission spectra were recorded over the time. The results showed that unlike **SC‐A** (Figure 2B) and **SC‐T** (Figure S2) that slowly photobleached with no sign of conversion, **SCs** bearing ASORMs, namely **SC‐F** (Figure 2C) and **SC‐P** (Figure 2D), photoconverted rapidly towards a similar blue‐shifted emissive form (λ_Em_=486 and 483 nm respectively) with a hypsochromic shift of 24 nm and 68 nm, respectively (Table 1).

To further characterize the converted **SC** (**cSC**), HPLC/UV/Visible/mass spectrometry analysis were performed before and after irradiation (Figure 2E and S3–6). The results showed that both **SC‐P** and **SC‐F** converted towards isomers of oxidized forms (M+O_2_), which displayed blue‐shifted absorption spectra due to dearomatization of the ASORMs. In addition, ^1^H NMR of **SC‐P** showed that after irradiation only the signals corresponding to the pyrrole moiety were changed (Figure S7). According to these results a structure of **cSC‐P** where the pyrrole got oxidized in its pyrrolidinone form is proposed in Figure 2E, which is in line with reported photooxidized form of pyrrole.^[19]^ Interestingly, the photoproduct arising from **SCs** that did not photoconvert (**cSC‐A** and **cSC‐T**) also showed oxidized forms (M+nO_2_), along with cleaved photoproducts (Figure S8), thus demonstrating that Singlet Oxygen Reactive Moieties (ASORMs) preferentially direct the photooxidation towards themselves.

The similarity of both emission and excitation spectra between the final converted forms, **cSC‐P** and **cSC‐F** and the model of conversion **SC‐V** (Figure 2F, Table 1), suggested that the conversion arose from the disruption of the conjugation between the coumarin and the ASORMs.

To prove that this process involved singlet oxygen, a set of experiments was conducted on **SC‐P**. First, we showed that **SC‐P** was stable over the time at ambient conditions in absence of light (Figure S9). Then under irradiation in argon‐degassed methanol the kinetics of photoconversion was significantly decreased (Figure S10). The use of DPBF proved that **SC‐P** generated ^1^O_2_ upon light irradiation (Figure S11). Additionally, when ^1^O_2_ was independently generated through a 640 nm excited aluminum phthalocyanine, the conversion occurred within 10 s (Figure S12), proving that the conversion arose from the chemical reaction between ^1^O_2_ and **SC‐P** at its ground state. As **SC‐P** could generate other ROS than ^1^O_2_, the reaction of different ROS without light irradiation was also evaluated and showed that only hydroxyl radical (HO⋅) could also trigger the conversion (Figure S13). However, the use of HPF as HO⋅ probe showed that **SC‐P** does not generate HO⋅ under irradiation (Figure S14).

From those results, and knowing the photophysical properties of the **SCs**, their converted form (**cSCs**) similar to our model **SC‐V**, and by monitoring the decrease of the fluorescence signal over the time (Figure S15), several photophysical constants involved in the phototransformation have been determined and are reported in Table 1. Although **SC‐F** displayed an impressive estimated chemical conversion yield (η=97±9%) compared to **SC‐P** (η=30±1%), the latter possesses more interesting photoconversion properties with a higher quantum yield of phototransformation (φ_Pt_) and photoconversion (φ_Pc_), depicting a higher photoreactivity (100‐fold compared to **SC‐A**), and a higher spectral shift upon conversion of 68 nm (24 nm for **SC‐F**), which is preferable for bioimaging applications. Interestingly, the quantum yields of photobleaching (φ_Bl_) of the converted forms (**cSC‐P** and **cSC‐F**) were shown to be slightly lower than for **SC‐V** and for non‐convertible **SCs**, thus depicting photostable photoproducts after conversion (Table 1).

Overall these sets of experiments confirmed our hypothesis that efficient dual‐emissive photoconvertible probes can be obtained through DPIC mechanism and a detailed mechanism is proposed in Figure 3. Upon irradiation in the visible range **SC‐P** reaches an excited state that can transfer its energy to triplet oxygen (^3^O_2_) to generate singlet oxygen (^1^O_2_) through intersystem crossing and deexcitation of the triplet state to the ground state. In the presence of the generated ^1^O_2_, **SC‐P** undergoes phototransformations (characterized by Φ_Pt_). Indeed, at its ground state **SC‐P** reacts with ^1^O_2_ either on the core coumarin leading to photobleaching (characterized by Φ_Bl_) or in a directed manner towards the ASORM resulting in photoconversion (characterized by Φ_Pc_ and the chemical yield η) and leading to a blue‐shifted fluorescent dye (**cSC‐P**) due to the disruption of the conjugation between the coumarin and the ASORM. Other key parameters include the fluorescence brightness of both the initial probe (ϵ and Φ_F_) and its converted form (ϵ_c_ and Φ_Fc_), as well as their quantum yield of ^1^O_2_ generation (Φ_Δ_).

**Figure 3:**
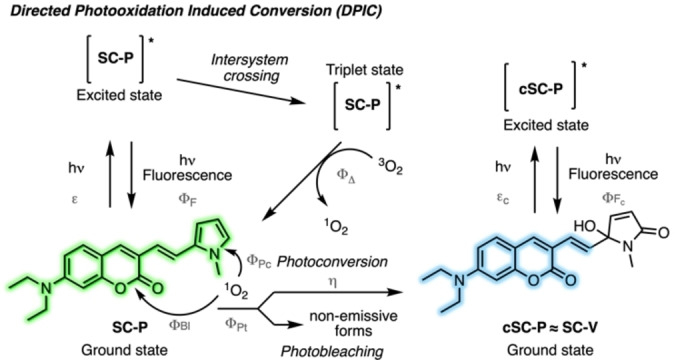
Proposed mechanism for the Directed Photooxidation Induced Conversion. ^1^O_2_ is generated upon irradiation and reacts at the ground state leading to a phototransformation (φ_Pt_) which can be photoconversion (φ_Pc_) or photobleaching (φ_Bl_). The constants (in grey) were determined (Table 1) and support the presented mechanism (for definitions see Supporting Information). Φ_Δ_ could not be determined due to the competitive reactivity of **SCs** and DPBF towards ^1^O_2_ (See Figure S11).

To demonstrate the application of DPIC mechanism in bioimaging, **SC‐P**, which displayed the fastest conversion along with the highest shift in emission, was chosen for cellular imaging in Hela cells (Figure 4). Prior to cell experiments, cytotoxicity and phototoxicity assays were performed and showed that **SC‐P** was not cytotoxic at 1 μM and not phototoxic after that the photoconversion was performed (Figure S16). As shown in Figure 4A, conversion was successfully achieved after one frame as the intensity of **SC‐P** and **cSC‐P** were respectively decreased and increased upon high irradiation at 488 nm (Figure 4B) with an average conversion yield of 91±8%. Advantageously, the conversion can be triggered at a desired timepoint (Figure S17) as well as in various selected regions of interest without converting the surrounding regions (Figure S18), showing a good spatiotemporal control of the conversion. Moreover, **cSC‐P** was sufficiently stable to be tracked over time (at 1 Hz) over several frames (Figure S17, S18). Using SMCy5.5 as a counterstain^[21]^ (Figure 4C), we then showed that **SC‐P** unexpectedly label lipids droplets (LDs) with a high association rate with SMCy5.5 of 83%, probably due to its non‐charged and relatively lipophilic nature (cLogP=2.97). Importantly, the converted form was shown to keep the ability to remain in LDs after conversion as **cSC‐P** association rate with **SC‐P** reached 94% (Figure 4D). Additionally, taking advantage of MemBright‐labeled endosomes,^[22]^ we checked that **SC‐P** was not endocytosed. Indeed, **SC‐P** was very weakly associated with endosomes (1.38%, Figure S19). Finally, we proved that both **SC‐P** and **cSC‐P** could be tracked over 50 frames (Figure 4E), and shared the same trajectories and average speed than SMCy5.5 upon tracking (Figure 4F). Overall, these experiments demonstrated that **SC‐P** and its readily obtained photoconverted form **cSC‐P**, preferentially stains the LDs and can be both tracked over large spatiotemporal scales in bioimaging.

**Figure 4:**
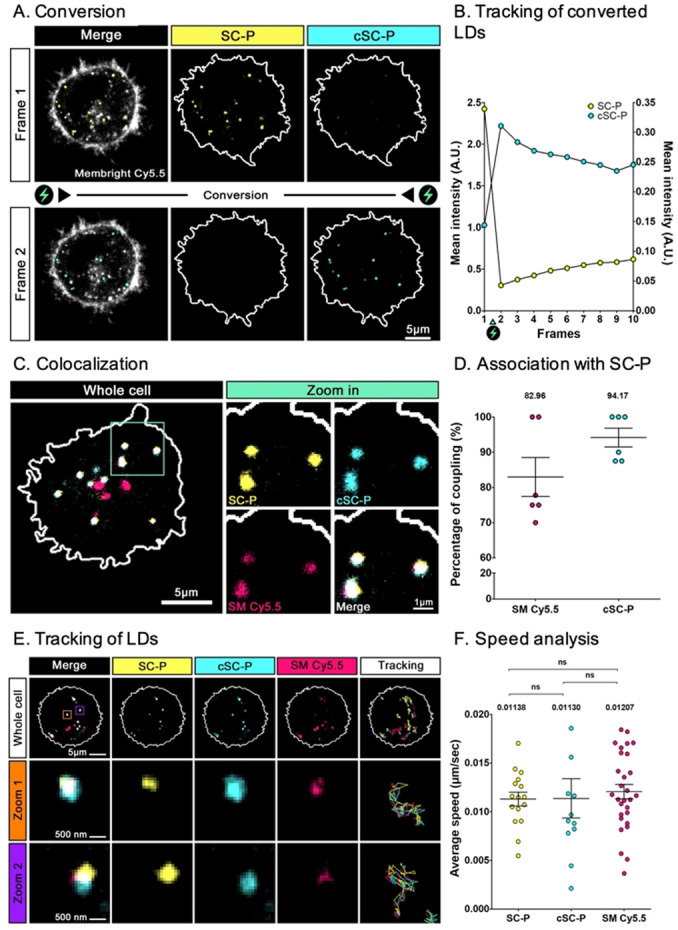
**SC‐P** labels LDs in Hela cells and can be converted and tracked using confocal imaging. (A) Hela Cells were labelled with **SC‐P** (1 μM, yellow) and counter stained for plasma membrane and endosomes with MemBright Cy5.5 (grey). Conversion of **SC‐P** (frame 1) toward **cSC‐P** (in cyan) can be induced with a 488 nm laser stimulation (green flash) and tracked over time. (B) Evolution of mean fluorescence intensity of **SC‐P** and **cSC‐P** after conversion (1 frame per second). (C) Colocalization analysis showed strong association of **SC‐P** (yellow), **c‐SC‐P** (cyan) with LDs probe SMCy5.5 (Magenta). (D) Coupling analysis of **SC‐P** shows strong association with SMCy5.5 (82,66%, p value <10^−38^) or **cSP‐P** (94,17% p value <10^−171^) on 6 independent cells (*n*=73 SMCy5.5 spots, and *n*=54 **c‐SC‐P** spots). (E) Tracking over time of SMCy5.5 (magenta) with **SC‐P** (yellow) and **cSC‐P** (cyan) shows correlated trajectories of vesicles over 50 frames. (F) Speed analysis of tracked vesicles reveals no statistical differences between lipid droplets vesicles (SMCy5.5) and **SC‐P**/**cSC‐P** spots.

In conclusion, we presented a new concept based on the Directed Photooxidation Induced Conversion (DPIC) mechanism enabling to obtain bright fluorophores that readily photoconvert upon visible light towards photostable blue‐shifted photoproducts. Although furan was found to provide a cleaner conversion, pyrrole, due to its electron rich nature was found to be a more suitable Aromatic Singlet Oxygen Reactive Moiety (ASORM), as it provided an important emission shift once conjugated to the fluorophore and displayed faster conversion rate. This approach led to **SC‐P**, a coumarin‐based DCPF that was successfully used to photoconvert and track LDs in live cells. Preliminary results from our group suggest that substituted pyrroles provide conversion as well and that this method could be applied to other fluorophores, opening opportunities to develop new efficient DCPFs and paving the way to new photoconvertible fluorescent probes for advanced microscopy.

## Conflict of interest

The authors declare no conflict of interest.

## Supporting information

As a service to our authors and readers, this journal provides supporting information supplied by the authors. Such materials are peer reviewed and may be re‐organized for online delivery, but are not copy‐edited or typeset. Technical support issues arising from supporting information (other than missing files) should be addressed to the authors.

Supporting Information

## Data Availability

The data that support the findings of this study are available from the corresponding author upon reasonable request.
