# Supplementary material for: Dual‐Color Photoconvertible Fluorescent Probes Based on Directed Photooxidation Induced Conversion for Bioimaging
Source: Angew Chem Int Ed Engl. 2022 Dec 14;62(4):e202215085. doi: 10.1002/anie.202215085 (PMC10107923; doi:10.1002/anie.202215085)
Supplement: Supplementary file 1 — Supporting Information [file ANIE-62-0-s001.pdf]

Angewandte Chemie
Eine Zeitschrift der Gesellschaft Deutscher Chemiker

Supporting Information

Dual-Color Photoconvertible Fluorescent Probes Based on Directed Photooxidation Induced Conversion for Bioimaging

*L. Saladin, V. Breton, O. Dal Pra, A. S. Klymchenko, L. Danglot, P. Didier, M. Collot**

Supplementary information

1) **Protocol and Synthesis .... p 1**
2) **Materials and methods .... p 21**
3) **Spectroscopic studies .... p 24**
4) **Analysis of photoproducts .... p 26**
5) **$^1O_2$ dependency .... p 32**
6) **Fluorescence decay curves .... p 34**
7) **Cytotoxycity phototoxicity assays .... p 35**
8) **Fluorescence microscopy studies .... p 36**
9) **References.. p.38**

1) Protocol and Synthesis

Synthesis of aldheyde 4

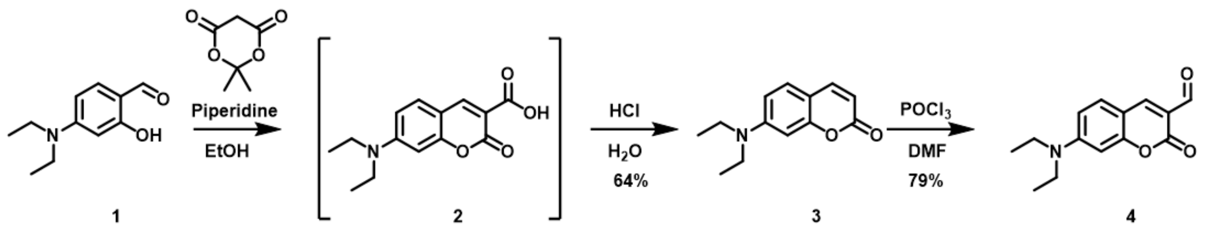

1 2 3 4

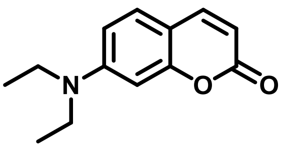

3. To a solution of 4-(Diethylamino)salicylaldehyde **1** (2.00 g, 10.35 mmol, 1 eq) and Meldrum's acid (1.64 g, 11.38 mmol, 1.1 eq) in EtOH (15 mL) was added piperidine (0.1 mL, 1.03 mmol, 0.1 eq,). The solution was warmed at 90°C overnight, cooled down with ice bath. The orange solid **2** was filtered, added to a solution of HCl 17%. The solution was refluxed at 130°C until disappearance of the suspension. The solution was cooled on ice bath and neutralized with NaOH (6M). The precipitate was filtered and washed with water to give **3** as a yellow solid (1.44 g, 64%). The NMR was in accordance with the literature.[1] ^{1}H NMR (400 MHz, $CDCl_3$) δ 7.53 (dd, *J* = 9.3, 0.6 Hz, 1H, H Ar), 7.26 (s, 1H, H Ar), 7.24 (d, *J* = 8.8 Hz, 1H, H Ar), 6.56 (dd, *J* = 8.8, 2.5 Hz, 1H, H Ar), 6.49 (d, *J* = 2.4 Hz, 1H, H Ar), 6.03 (d, *J* = 9.3 Hz, 1H), 3.41 (q, *J* = 7.1 Hz, 4H, CH_2 ethyl), 1.21 (t, *J* = 7.1 Hz, 6H, CH_3 ethyl).

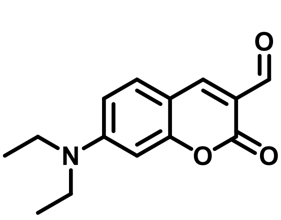

4. To a solution of DMF (2 mL, 26 mmol, 4 eq) was dropwise added $POCl_3$ (3.28 g, 21 mmol, 3.3 eq, 2 mL) at 0°C under argon. The solution was stirred 20 minutes at 60°C. **3** (1.4 g, 6.44 mmol) in 10 mL of DMF was added and the reaction was heated at 60°C for 3 hours. The reaction was followed by TLC. After the disappearance of the starting material the mixture was cooled down and poured into water. The precipitate was filtered and then solubilized in DCM, washed with a solution of saturated $NaHCO_3$, dried over $MgSO_4$. Solvents were evaporated under reduced pressure to give **4** as an orange solid (1.25 g, 79%). The NMR was in accordance with the literature.[2] ^{1}H NMR (400 MHz, $CDCl_3$) δ 10.12 (s, 1H, CHO), 8.25 (d, *J* = 0.7 Hz, 1H, H Ar), 7.41 (d, *J* = 9.0 Hz, 1H, H Ar), 6.63 (dd, *J* = 9.0, 2.5 Hz, 1H, H Ar), 6.48 (dd, *J* = 2.5, 0.7 Hz, 1H, H Ar), 3.47 (q, *J* = 7.2 Hz, 4H, CH_2 ethyl), 1.25 (t, *J* = 7.1 Hz, 6H, CH_3 ethyl).

Synthesis of phosphonium 7

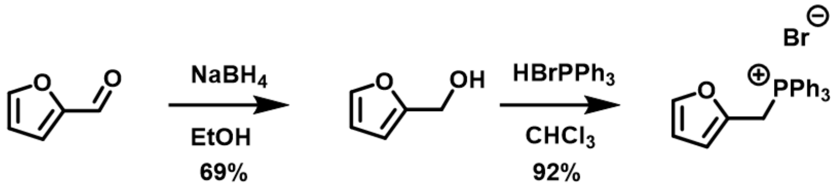

5 6 7

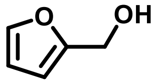

6. To a solution of furfural **5** (1 g, 10.4 mmol, 1 eq, 0.86 mL) at 0°C in ethanol (10 mL) was added dropwise $NaBH_4$ (393 mg, 10.4 mmol, 1 eq). The solution was allowed to warm at RT. Acetone (0.1 mL) and water (10 mL) were added to the mixture. The product was extract with DCM, washed with brine, and dried over $MgSO_4$. Solvents were evaporated under reduced pressure to give **6** (700 mg, 69%). The NMR was in accordance with the literature except for the hydroxyl proton which is prompt to shift.[3] ^{1}H NMR (400 MHz, $CDCl_3$) δ 7.40 (dd, J = 1.9, 0.8 Hz, 1H), 6.34 (dd, J = 3.2, 1.9 Hz, 1H), 6.31 – 6.26 (m, 1H), 4.61 (d, J = 5.7 Hz, 2H), 1.91 – 1.78 (m, 1H).

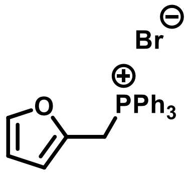

7. To a solution of $HBrPPh_3$ (1.31 g, 3.81 mmol, 1.1 eq) in ACN (10 mL) was added **6** (340 mg, 3.47 mmol, 1 eq). The solution was refluxed 2 hours. The mixture was cooled down at RT and filtered. The powder was washed with Et_2O to give **7** (1.35 g, 92%) as an off-white powder. The NMR was in accordance with the literature.[4] ^{1}H NMR (400 MHz, $CDCl_3$) δ 7.77 (dt, J = 12.2, 7.0 Hz, 9H), 7.67 – 7.62 (m, 6H), 7.18 (s, 1H), 6.57 (d, J = 3.9 Hz, 1H), 6.25 (s, 1H), 5.64 (d, J = 13.1 Hz, 2H).

Synthesis of SC via the Wittig reaction

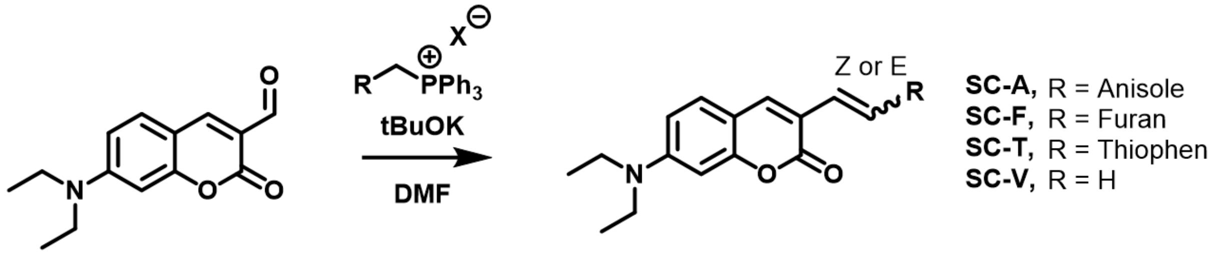

4

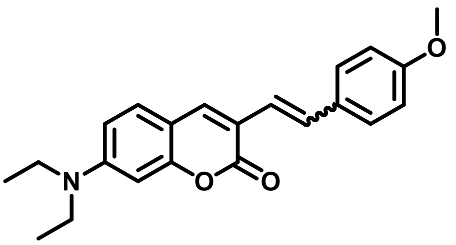

SC-A. To a solution of **4** (100 mg, 0.407 mmol, 1 eq) and (4-methoxybenzyl)triphenylphosphonium chloride (205 mg, 0.490 mmol, 1.2 eq) in DMF (10 mL) was added tBuOK (64 mg, 0.571 mmol, 1.4 eq). The reaction mixture was allowed to stir at RT for 5 minutes. The mixture was diluted with water and extracted with Et_2O. The organic phase was washed with saturated $NaHCO_3$ solution, then with brine solution, dried over $MgSO_4$, filtered and concentrated. The crude was purified by column chromatography on silica gel (DCM/Heptane: 40/60 to 100/0) to obtain **SC-A** (Z) as an orange amourphous solid (25 mg, 18%) and **SC-A** (E) as an orange amourphous solid (12.5 mg, 9%). $Rf_Z = 0.33$, $Rf_E = 0.44$ (DCM). **SC-A** (E): ^{1}H NMR (400 MHz, $CDCl_3$) δ 7.64 (s, 1H, ArH), 7.48 – 7.43 (m, 2H, ArH), 7.40 (d, J = 16.3 Hz, 1H, CH alkene), 7.29 – 7.25 (m, 1H, ArH), 6.96 (d, J = 16.3 Hz, 1H, CH alkene), 6.92 – 6.84 (m, 2H), 6.58 (dd, J = 8.8, 2.5 Hz, 1H), 6.50 (d, J = 2.5 Hz, 1H), 3.82 (s, 3H, OCH_3), 3.41 (q, J = 7.1 Hz, 4H, CH_2 ethyl),

1.21 (t, J = 7.1 Hz, 6H, CH_3 ethyl). ^{13}C NMR (101 MHz, $CDCl_3$) δ 161.58 (CO_2 Lactone), 159.35, 155.44, 150.23, 137.14, 130.47, 129.67, 128.62, 127.82, 120.98, 118.21, 114.13, 109.20, 109.06, 97.22, 55.32 (OMe), 44.85 (CH_2 ethyl), 12.51 (CH_3 ethyl). HRMS (ESI+) calculated for $C_{22}H_{24}NO_3$ $[M+H]^+$ 350.1756, found 350.1751. **SC-A** (Z): ^1H NMR (400 MHz, $CDCl_3$) δ 7.47 (d, J = 0.9 Hz, 1H, ArH), 7.31 – 7.23 (m, 2H, ArH), 7.01 – 6.94 (m, 1H, ArH), 6.80 – 6.72 (m, 2H, ArH), 6.55 (d, J = 12.3 Hz, 1H, CH alkene), 6.46 (d, J = 7.5 Hz, 2H, ArH), 6.38 (d, J = 12.3 Hz, 1H, CH alkene), 3.75 (s, 3H, OCH_3), 3.36 (q, J = 7.1 Hz, 4H, CH_2 ethyl), 1.16 (t, J = 7.1 Hz, 6H, CH_3 ethyl).^{13}C NMR (101 MHz, $CDCl_3$) δ 162.40 (CO_2 Lactone), 158.71, 155.99, 150.40, 140.34, 130.89, 129.94, 129.42, 128.84, 122.22, 117.29, 113.90, 108.80, 108.57, 97.29, 55.82 (OMe), 44.83 (CH_2 ethyl), 12.47 (CH_3 ethyl). HRMS (ESI+) calculated for $C_{22}H_{24}NO_3$ $[M+H]^+$ 350.1756, found 350.1784.

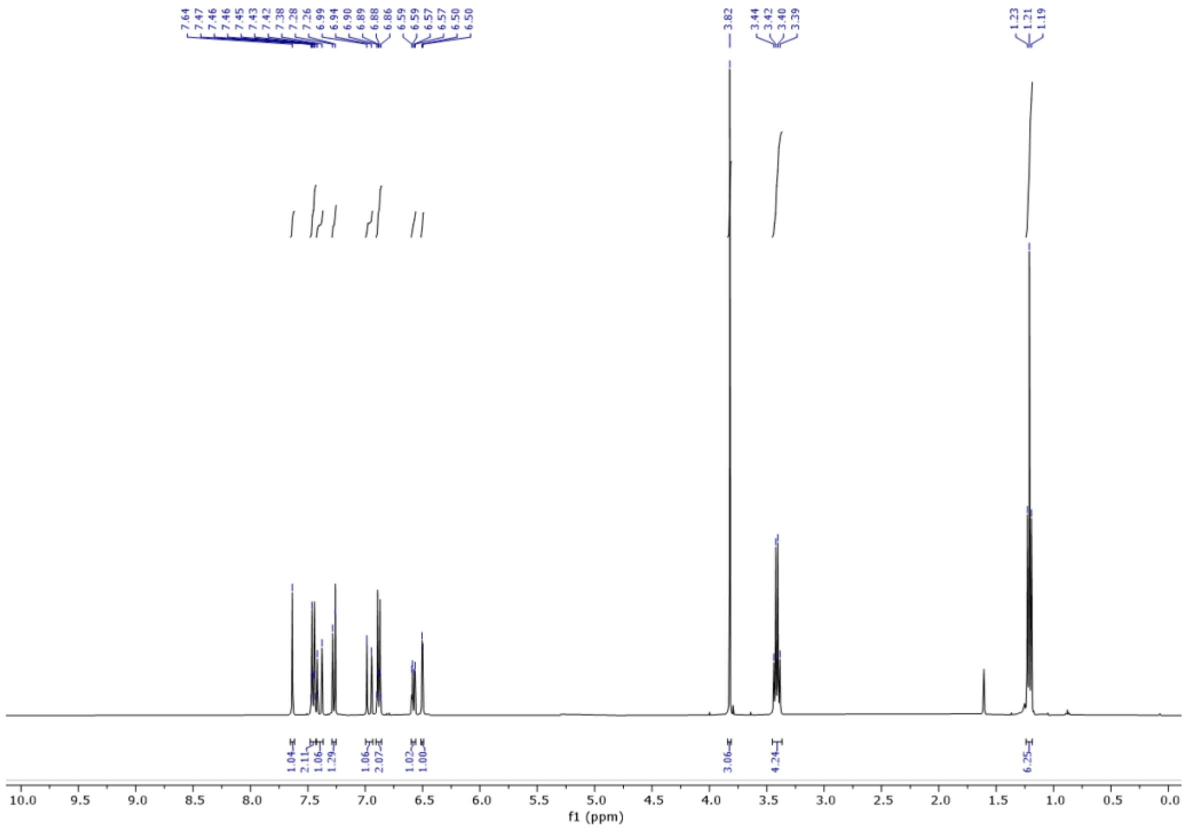

^1H NMR spectrum of **SC-A** (E)

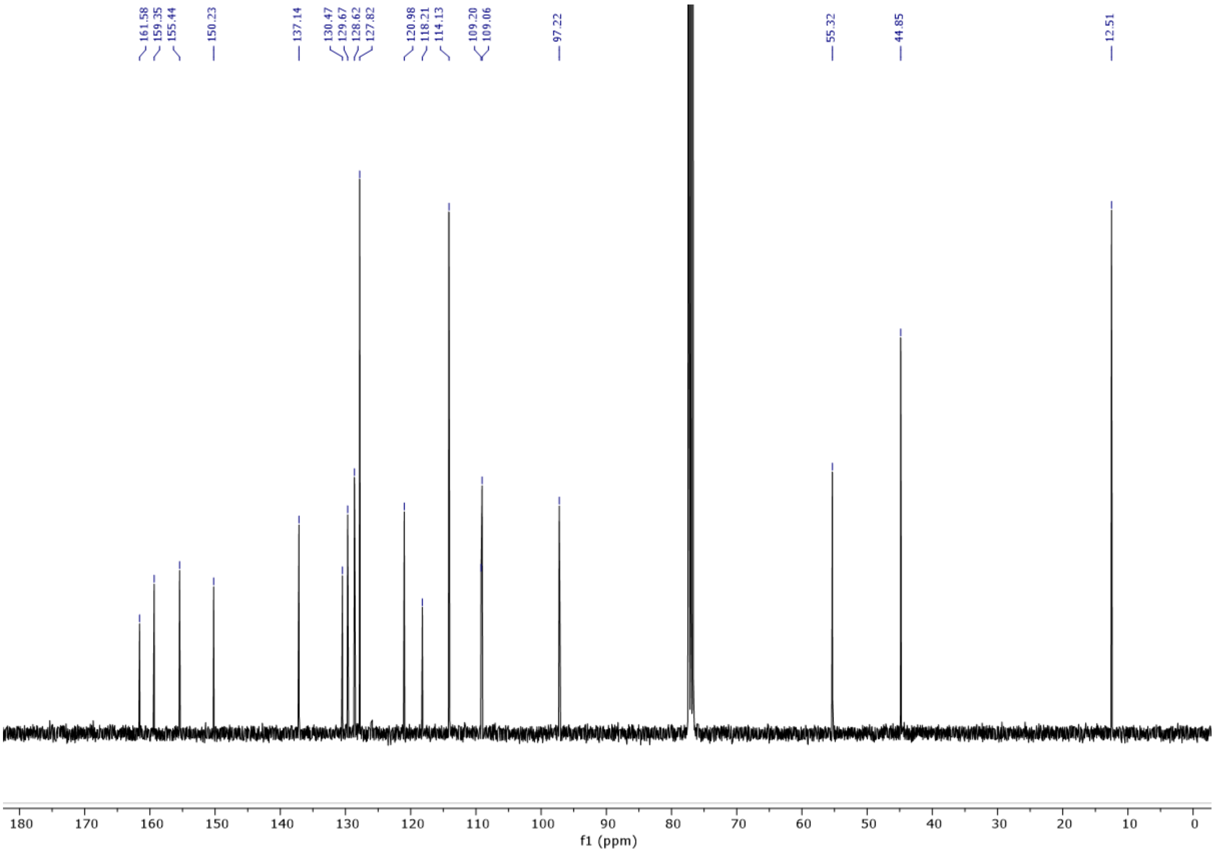

^{13}C NMR spectrum of **SC-A** (E)

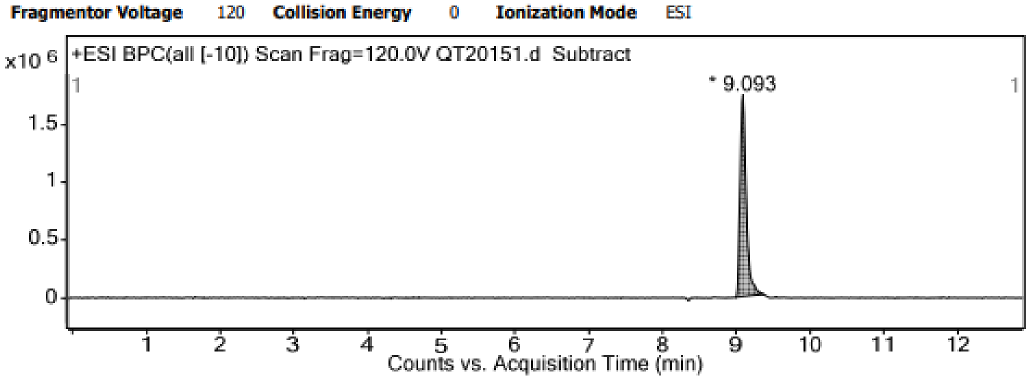

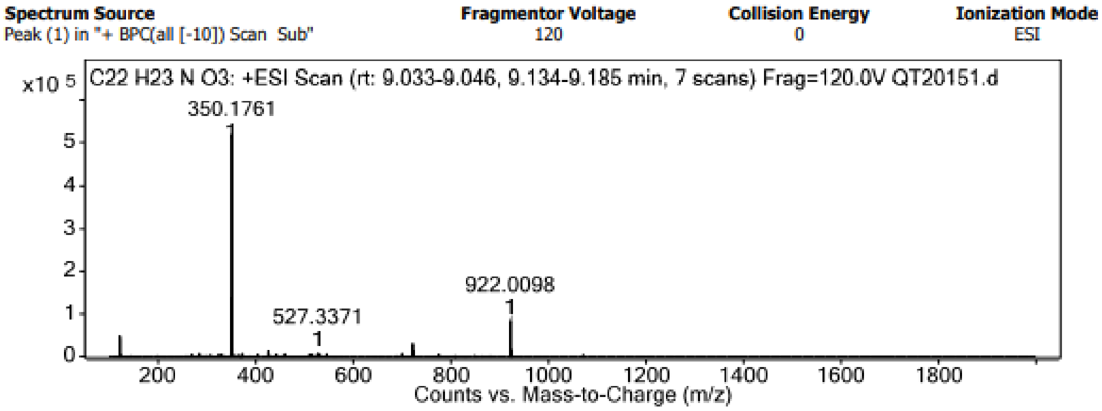

HRMS spectrum of **SC-A** (E)

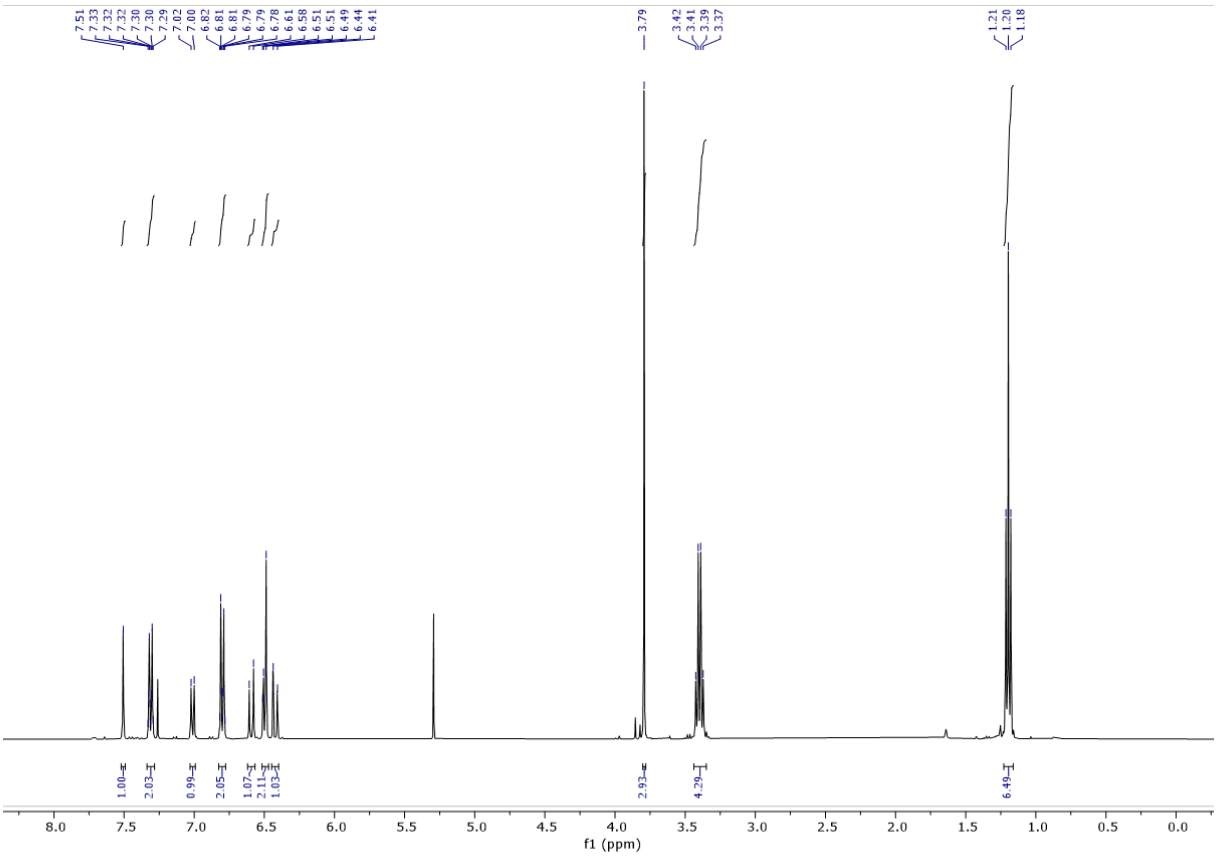

^{1}H NMR spectrum of **SC-A** (Z)

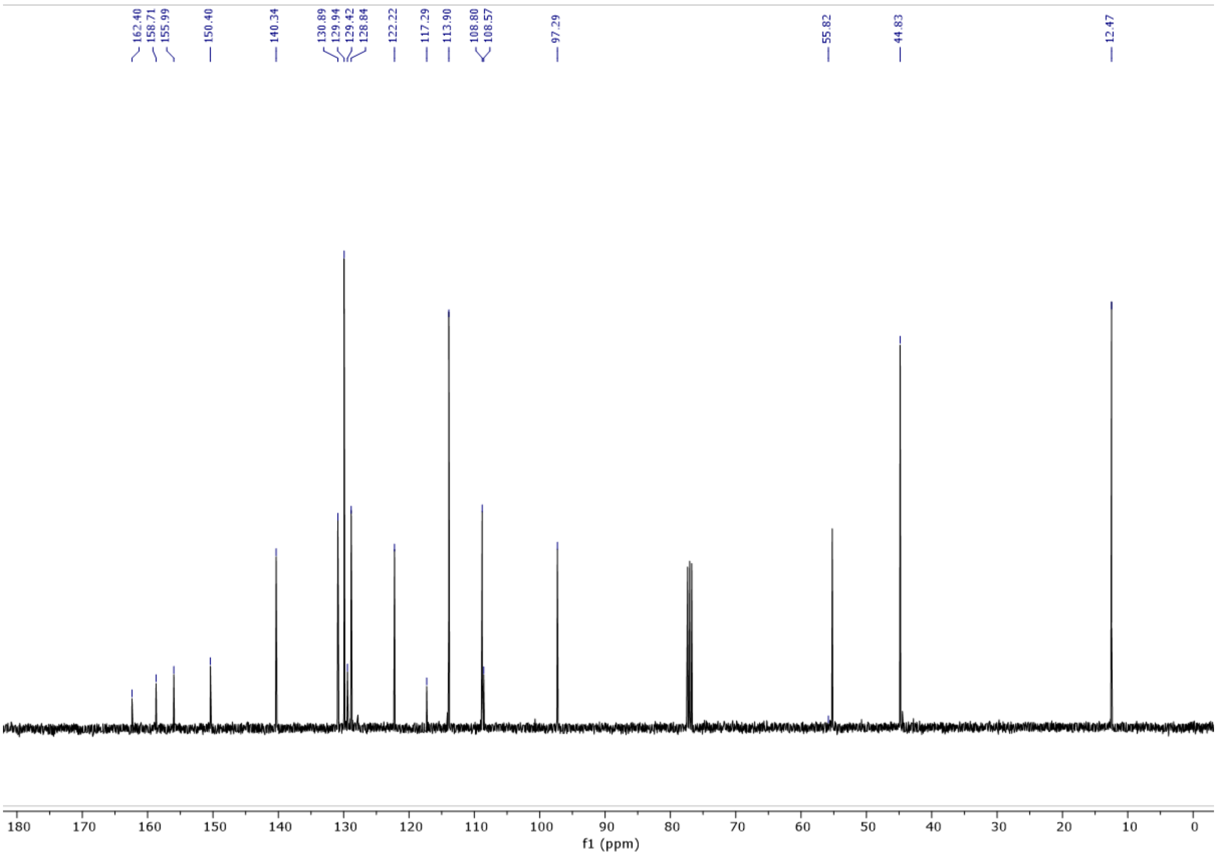

^{13}C NMR spectrum of **SC-A** (Z)

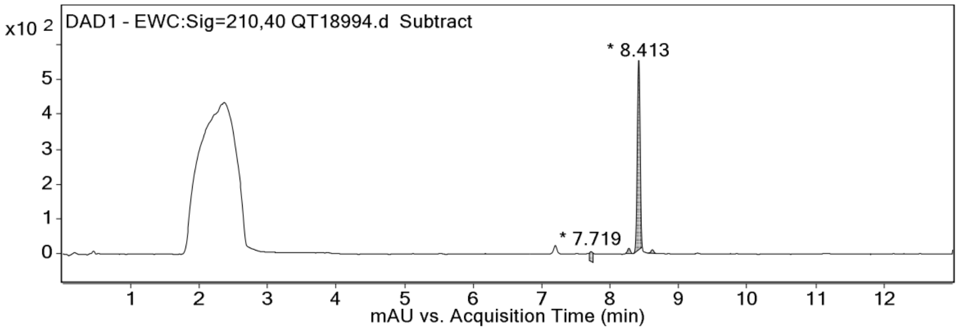

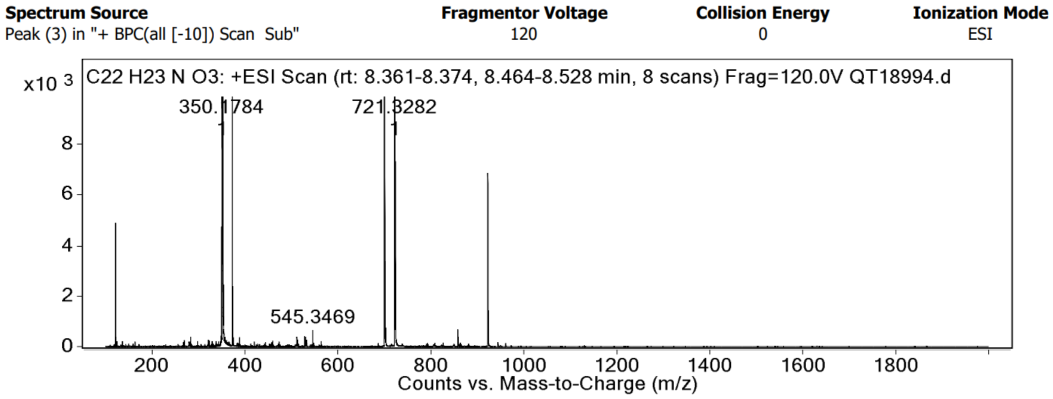

HRMS spectrum of **SC-A** (Z)

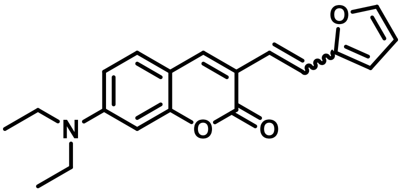

SC-F. To a solution of **4** (100 mg, 0.407 mmol, 1 eq) and **7** (207 mg, 0.489 mmol, 1.2 eq) in DMF (10 mL) was added tBuOK (64 mg, 0.571 mmol, 1.4 eq). The reaction mixture was allowed to stir at RT for 5 minutes. The mixture was diluted with water and extracted with Et_2O. The organic phase was washed with saturated $NaHCO_3$ solution, then with brine solution, dried over $MgSO_4$, filtered and concentrated. The crude was purified by column chromatography on silica gel (DCM/Heptane: 40/60 to 100/0) to obtain **SC-F** (E) as an orange amourphous solid (5 23mg, 4%) and **SC-F** (Z) as an orange amourphous solid (50 mg, 40%). Rf_E = 0.65, Rf_Z = 0.4 (DCM). **SC-F** (E): ^{1}H NMR (400 MHz, $CDCl_3$) δ 7.55 (s, 1H, ArH), 7.47 (d, J = 16.1 Hz, 1H, CH alkene), 7.39 (dd, J = 1.9, 0.6 Hz, 1H, ArH), 7.31 – 7.22 (d, J = 8 Hz, 1H, ArH), 6.92 (dd, J = 16.1 Hz, 1H, CH alkene), 6.58 (dd, J = 8.8, 2.5 Hz, 1H, ArH), 6.50 (d, J = 2.5 Hz, 1H, ArH), 6.41 (dd, J = 3.3, 1.8 Hz, 1H, ArH), 6.35 (dd, J = 3.4, 0.7 Hz, 1H, ArH), 3.42 (q, J = 7.1 Hz, 4H, CH_2 ethyl), 1.21 (t, J = 7.1 Hz, 6H, CH_3 ethyl). ^{13}C NMR (101 MHz, $CDCl_3$) δ 161.00 (CO_2 Lactone), 156.13, 153.69, 151.27, 142.17, 138.90, 128.70, 122.05, 118.62, 117.46, 112.79, 109.68, 109.07, 97.16, 44.86 (CH_2 ethyl), 13.03 (CH_3 ethyl). HRMS (ESI+) calculated for $C_{19}H_{20}NO_3$ $[M+H]^+$ 310.1443, found 310.1445. **SC-F** (Z): ^{1}H NMR (400 MHz, $CDCl_3$) δ 8.03 (s, 1H, ArH), 7.35 (t, J = 1.3 Hz, 1H, ArH), 7.27 (d, J = 8.8 Hz, 1H, ArH), 6.60 (dd, J = 8.8, 2.5 Hz, 1H, ArH), 6.53 (d, J = 2.4 Hz, 1H, ArH), 6.46 – 6.34 (m, 4H, ArH and CH alkene), 3.45 (q, J = 7.1 Hz, 4H, CH_2 ethyl), 1.24 (t, J = 7.1 Hz, 6H, CH_3 ethyl). ^{13}C NMR (101 MHz, $CDCl_3$) δ 159.86 (CO_2 Lactone), 156.14, 152.26, 148.90, 144.09, 140.79, 131.09, 120.85, 119.02, 116.57, 113.42, 111.30, 108.91, 108.71, 95.06, 48.78 (CH_2 ethyl), 13.44 (CH_3 ethyl). HRMS (ESI+) calculated for $C_{19}H_{20}NO_3$ $[M+H]^+$ 310.1443, found 310.1455.

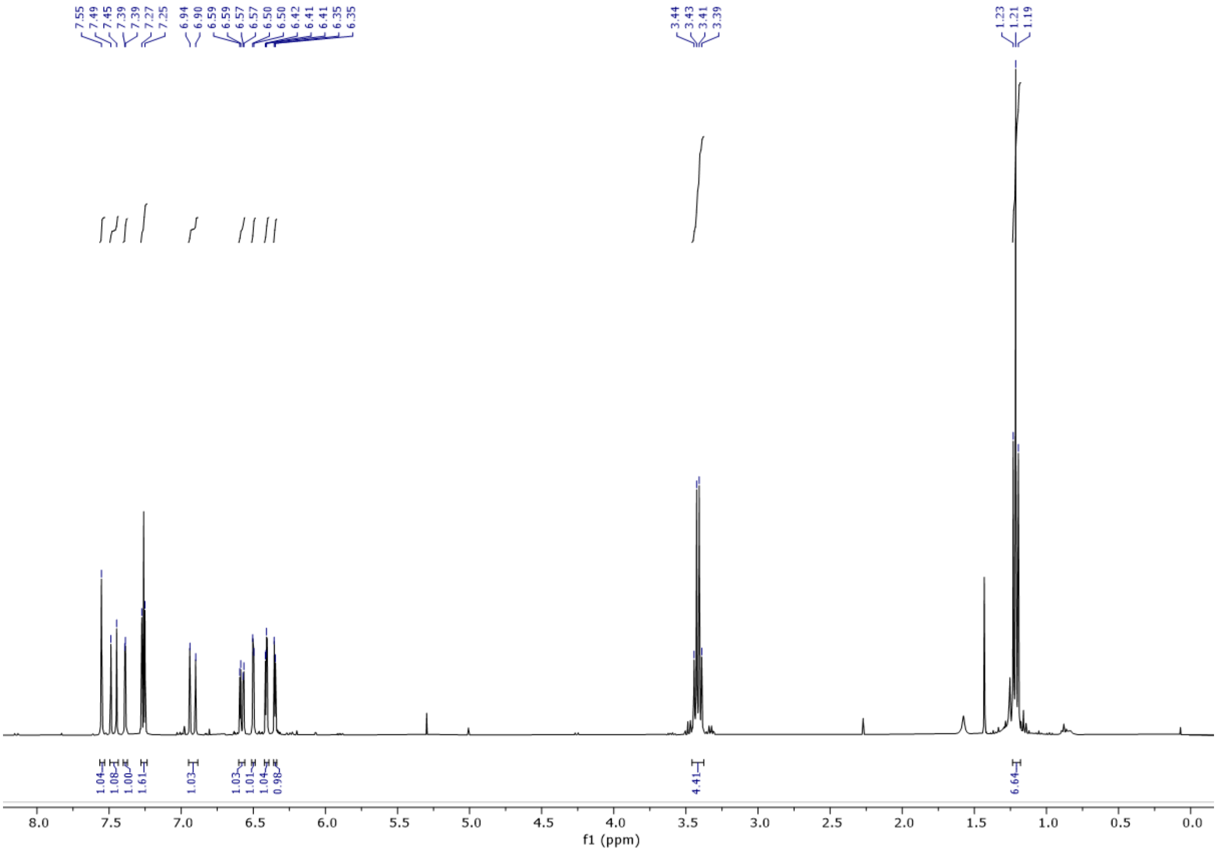

^{1}H NMR spectrum of **SC-F** (E)

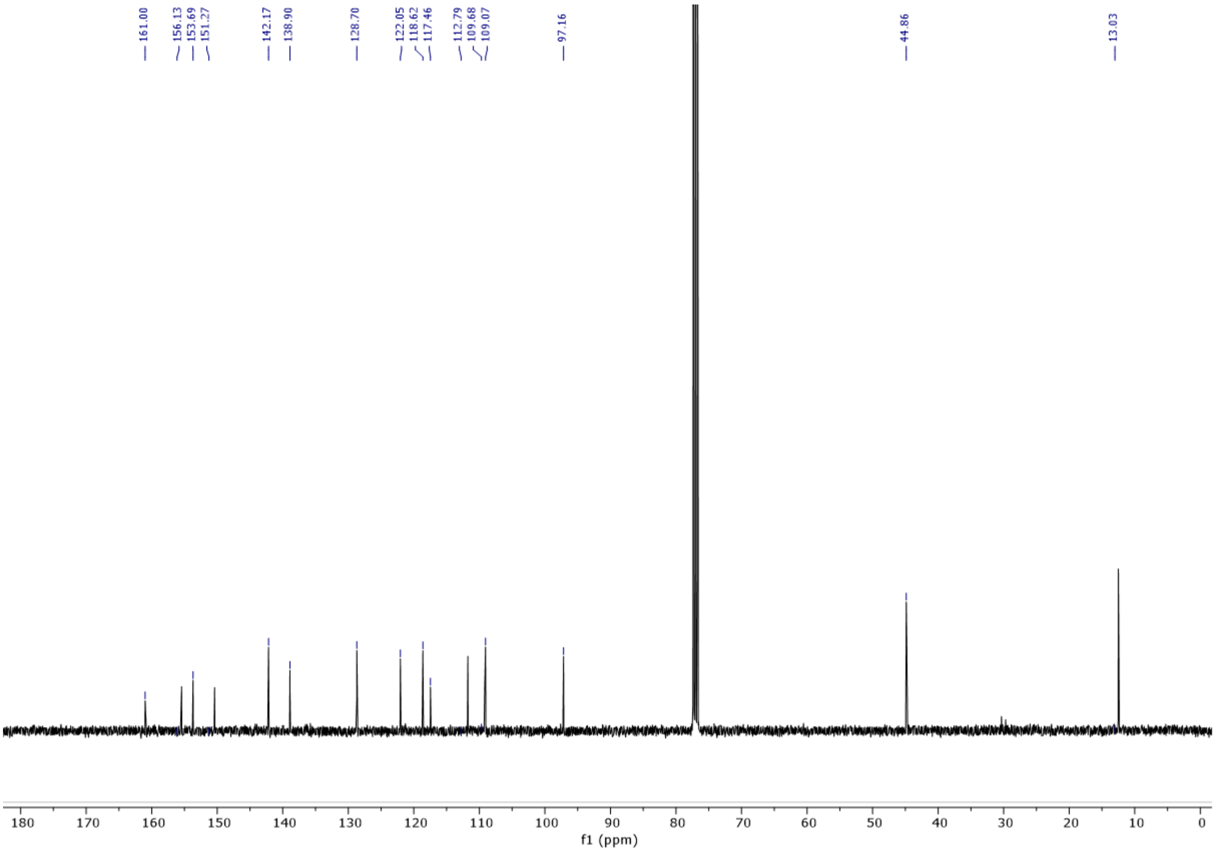

^{13}C NMR spectrum of **SC-F** (E)

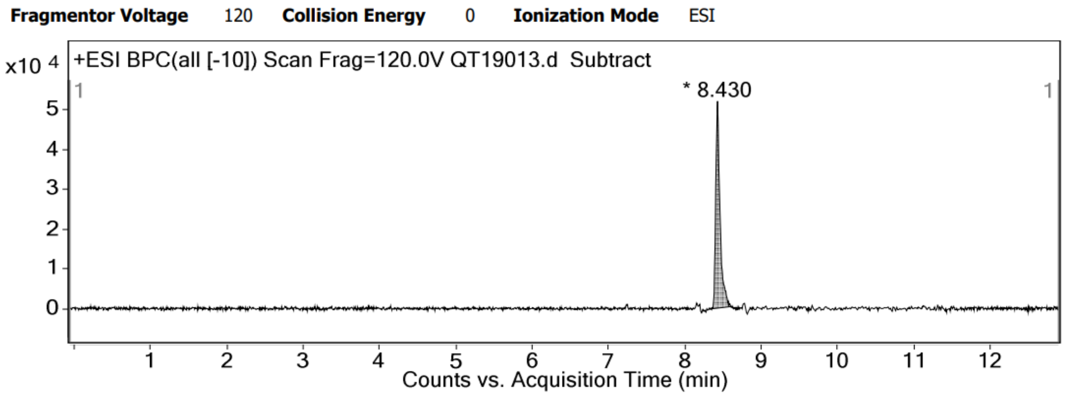

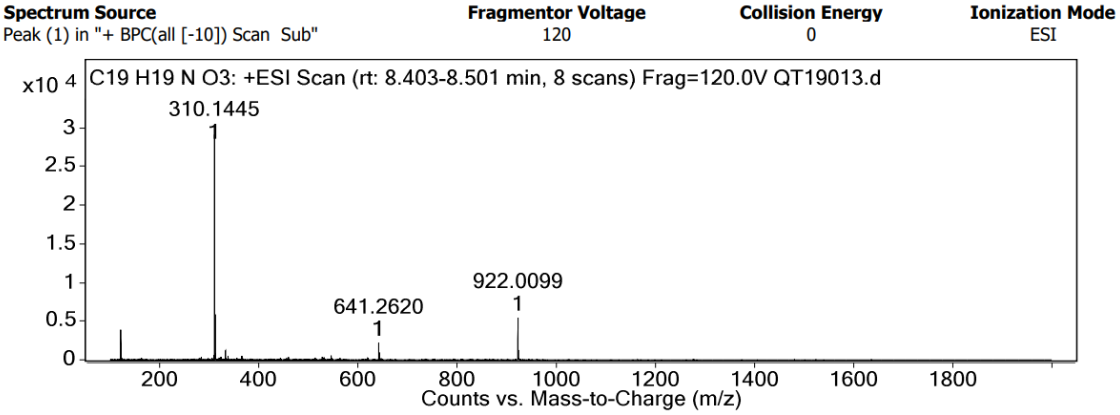

HRMS Spectrum of **SC-F** (E)

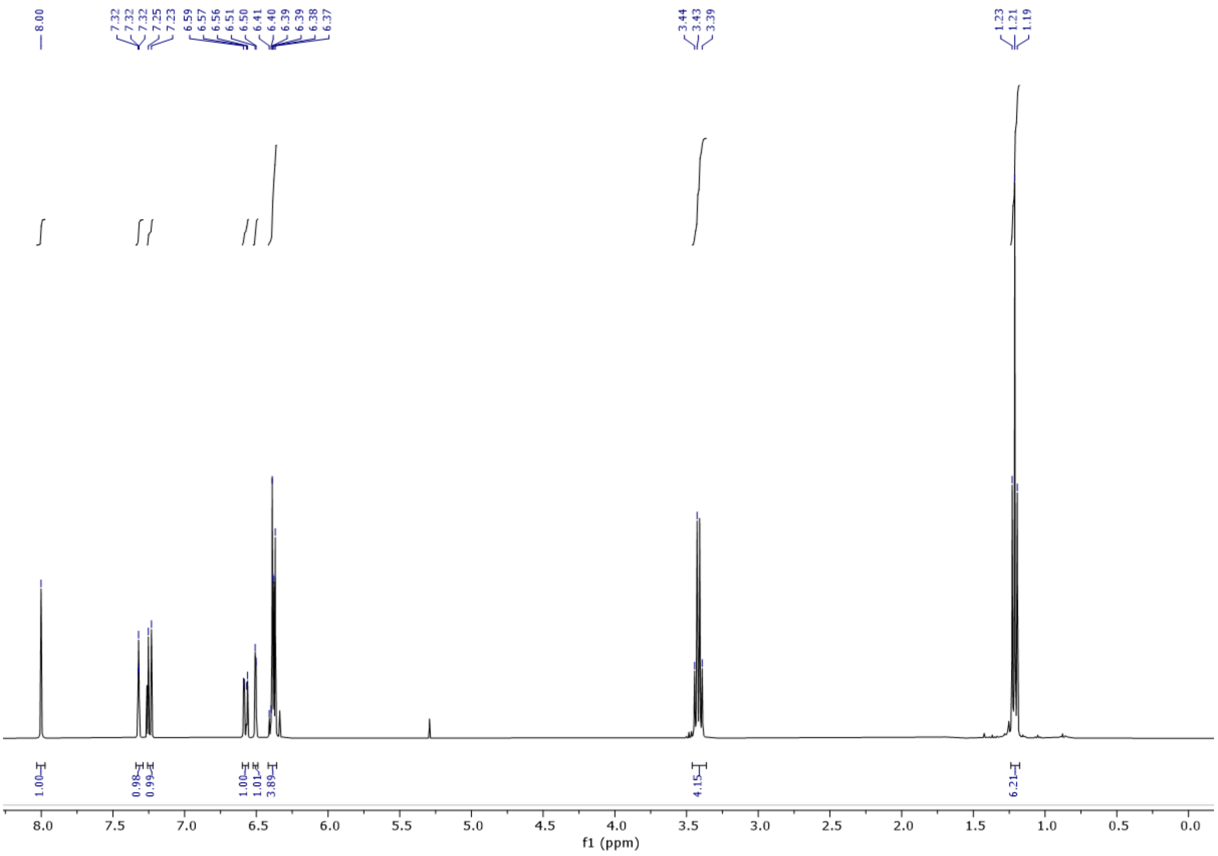

^{1}H NMR spectrum of **SC-F** (Z)

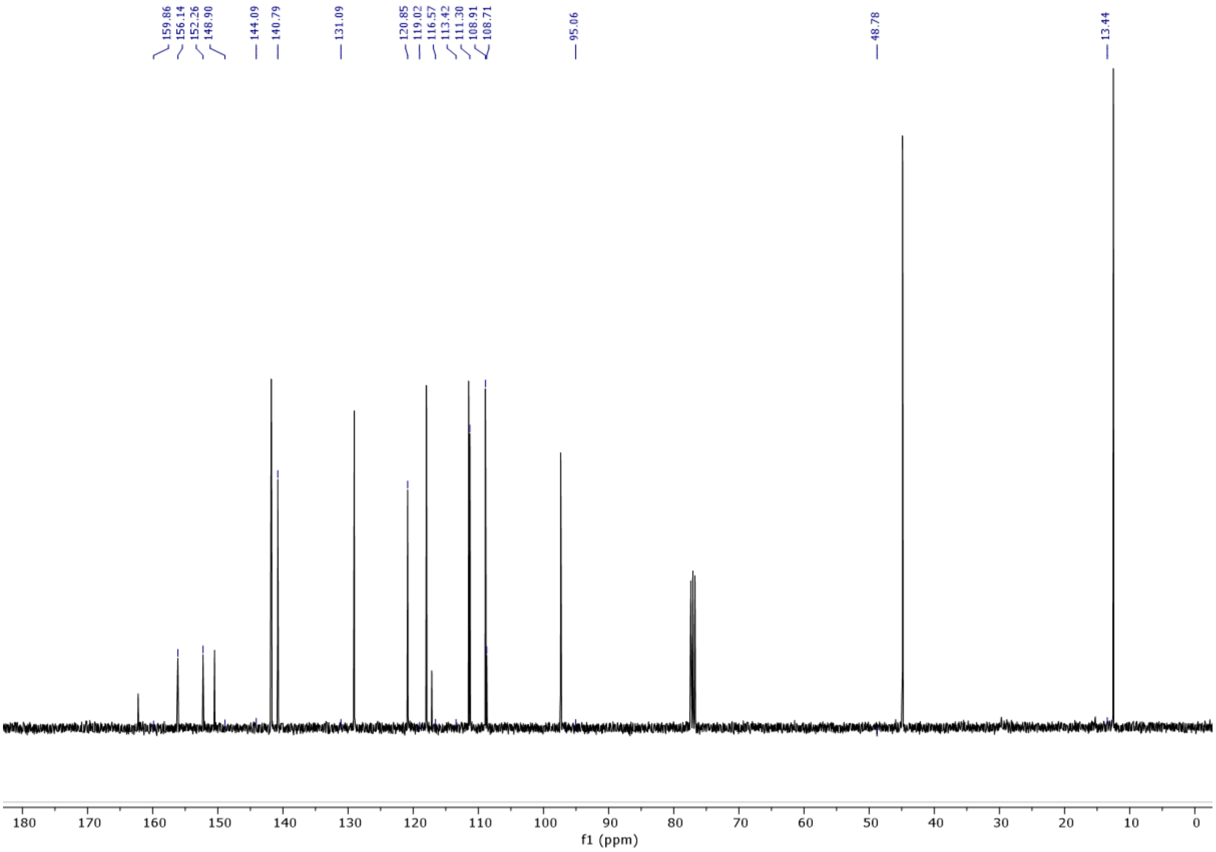

^{13}C NMR spectrum of **SC-F** (Z)

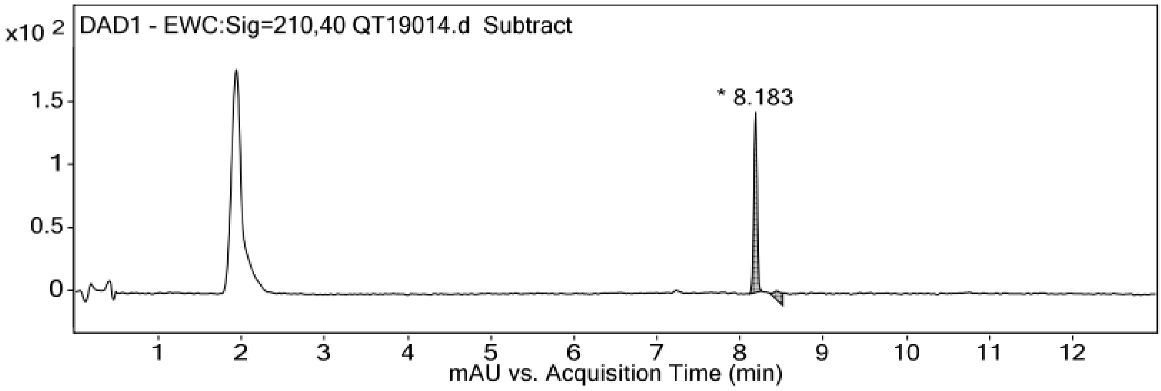

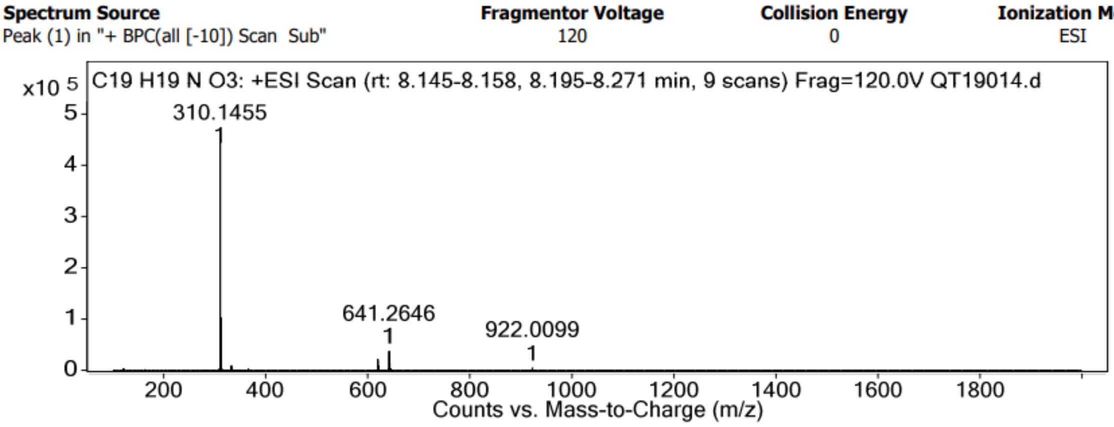

HRMS Spectrum of **SC-F (Z)**

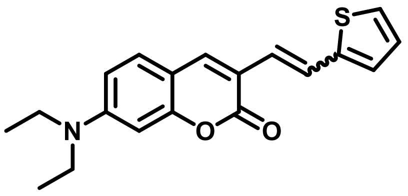

SC-T. To a solution of **4** (100 mg, 0.407 mmol, 1 eq) and Triphenyl(2-thienylmethyl)phosphonium bromide (215 mg, 0.490 mmol, 1.2 eq) in DMF (10 mL) was added tBuOK (64 mg, 0.571 mmol, 1.4 eq). The reaction mixture was allowed to stir at RT for 5 minutes. The mixture was diluted with water and extracted with Et_2O. The organic phase was washed with saturated $NaHCO_3$ solution, then with brine solution, dried over $MgSO_4$, filtered and concentrated. The crude was purified by column chromatography on silica gel (DCM) to obtain **SC-T** (Z) as an orange amorphous solid (90 mg, 68%) and **SC-T** (E) as an orange amorphous solid (5 mg, 4%). Rf_Z = 0.3, Rf_E = 0.63 (DCM). **SC-T** (E): ^1H NMR (400 MHz, $CDCl_3$) δ 7.55 (s, 1H, ArH), 7.47 (d, J = 16.1 Hz, 1H, CH alkene), 7.39 (dd, J = 1.9, 0.6 Hz, 1H, ArH), 7.30 – 7.23 (d, 1H, J = 8.8 Hz, ArH), 6.92 (d, J = 16.1 Hz, 1H, CH alkene), 6.58 (dd, J = 8.8, 2.5 Hz, 1H, ArH), 6.50 (d, J = 2.5 Hz, 1H, ArH), 6.41 (dd, J = 3.3, 1.8 Hz, 1H, ArH), 6.35 (dd, J = 3.3, 0.6 Hz, 1H, ArH), 3.42 (q, J = 7.1 Hz, 4H, CH_2 ethyl), 1.21 (t, J = 7.1 Hz, 6H, CH_3 ethyl). ^{13}C NMR (101 MHz, $CDCl_3$) δ 161.00 (CO_2 Lactone), 155.44, 153.69, 150.40, 142.17, 138.90, 128.70, 122.05, 118.62, 117.46, 111.76, 109.17, 109.10, 109.07, 97.16, 44.86 (CH_2 ethyl), 11.93 (CH_3 ethyl). HRMS (ESI+) calculated for $C_{19}H_{20}NO_2S$ $[M+H]^+$ 326.1215, found 326.1201. **SC-T** (Z): ^1H NMR (400 MHz, $CDCl_3$) δ 8.03 (s, 1H, ArH), 7.35 (t, J = 1.3 Hz, 1H, ArH), 7.27 (d, J = 8.8 Hz, 1H, CH alkene), 6.60 (dd, J = 8.8, 2.5 Hz, 1H, ArH), 6.53 (d, J = 2.4 Hz, 1H, ArH), 6.45 – 6.35 (m, 4H), 3.45 (q, J = 7.1 Hz, 4H, CH_2 ethyl), 1.24 (t, J = 7.1 Hz, 6H, CH_3 ethyl). ^{13}C NMR (101 MHz, $CDCl_3$) δ 162.23 (CO_2 Lactone), 156.14, 152.26, 150.50, 141.77, 140.79, 129.05, 120.85, 117.96, 117.15, 111.48, 111.30, 108.91, 108.71, 97.36, 44.85 (CH_2 ethyl), 12.08 (CH_3 ethyl). HRMS (ESI+) calculated for $C_{19}H_{20}NO_2S$ $[M+H]^+$ 326.1215, found 326.1199.

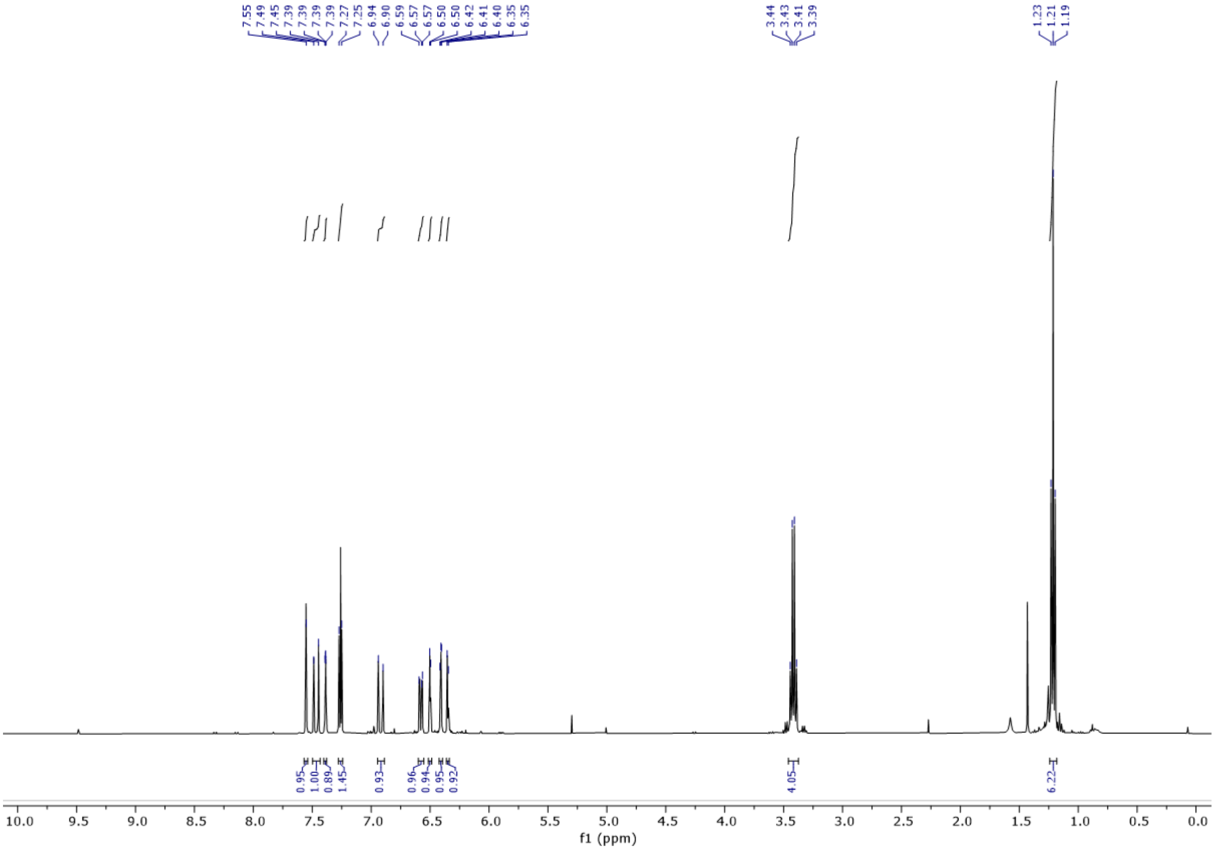

^1H NMR spectrum of **SC-T** (E)

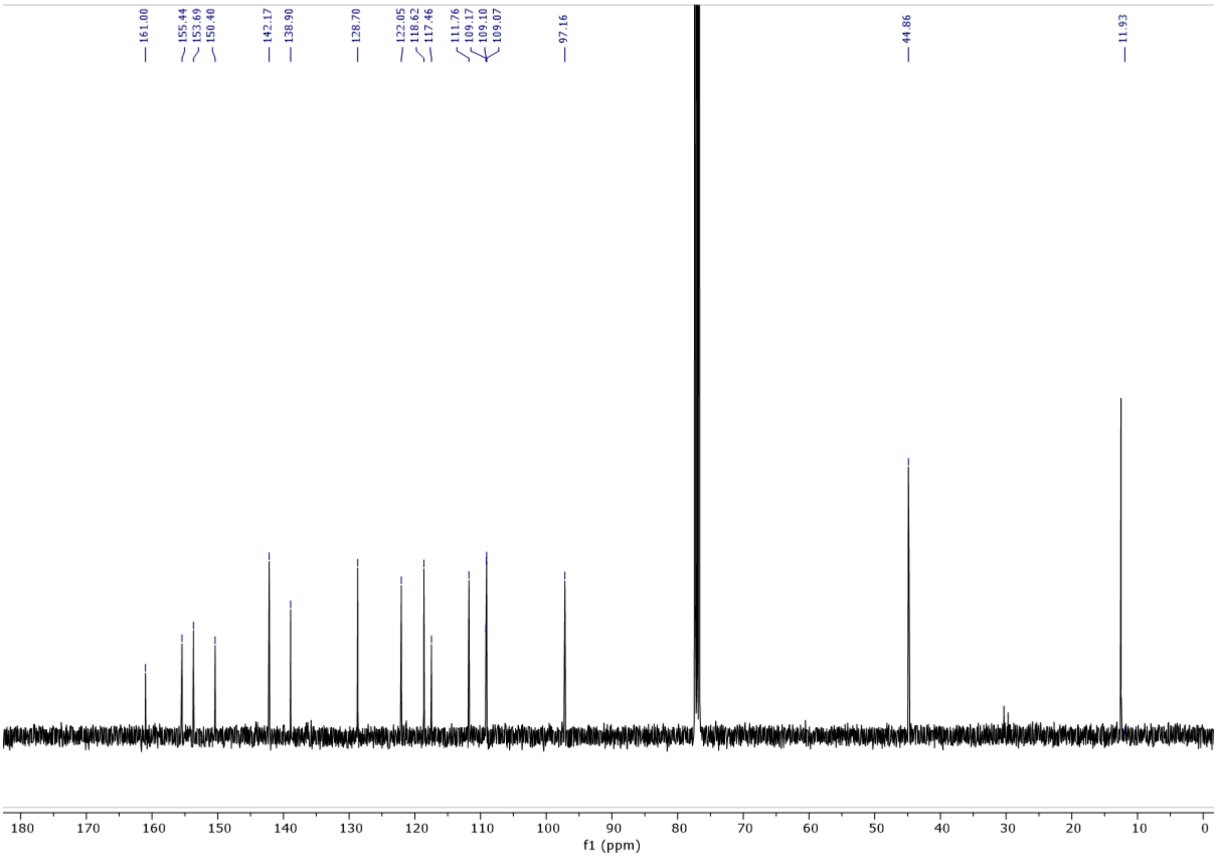

^{13}C NMR spectrum of **SC-T** (E)

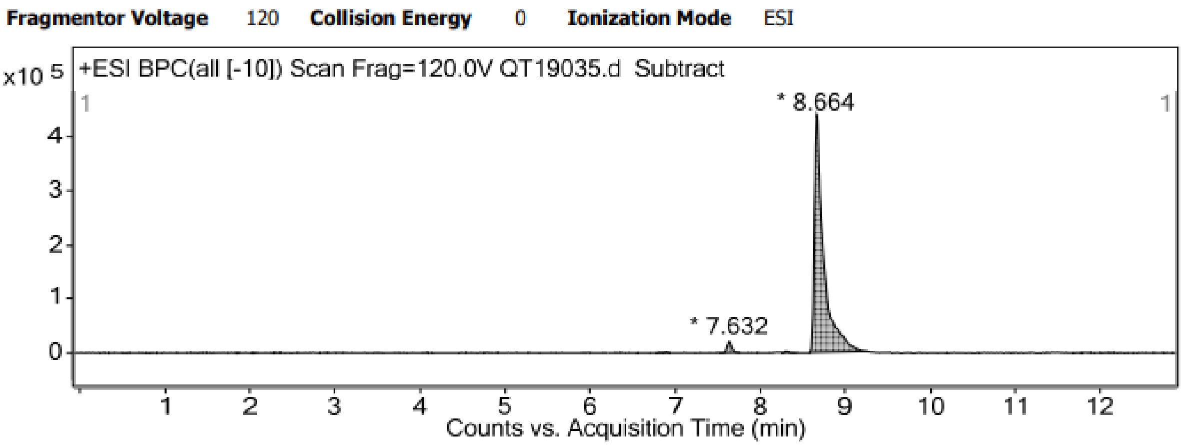

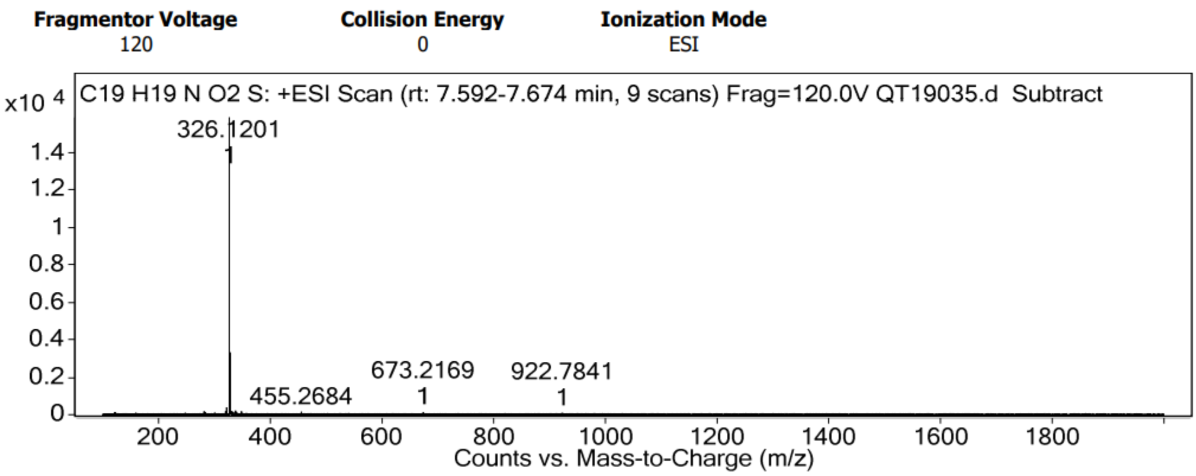

HRMS Spectrum of **SC-T (E)**

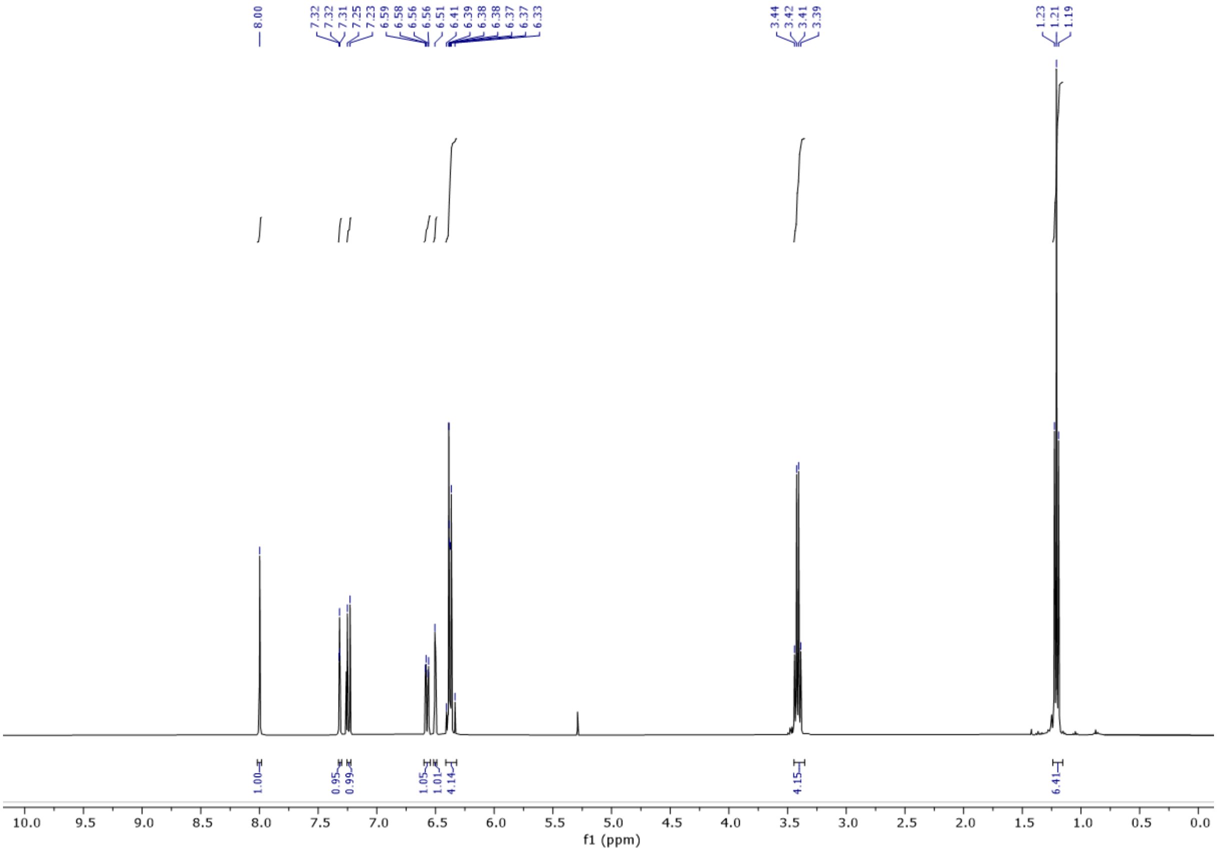

^{1}H NMR spectrum of **SC-T** (Z)

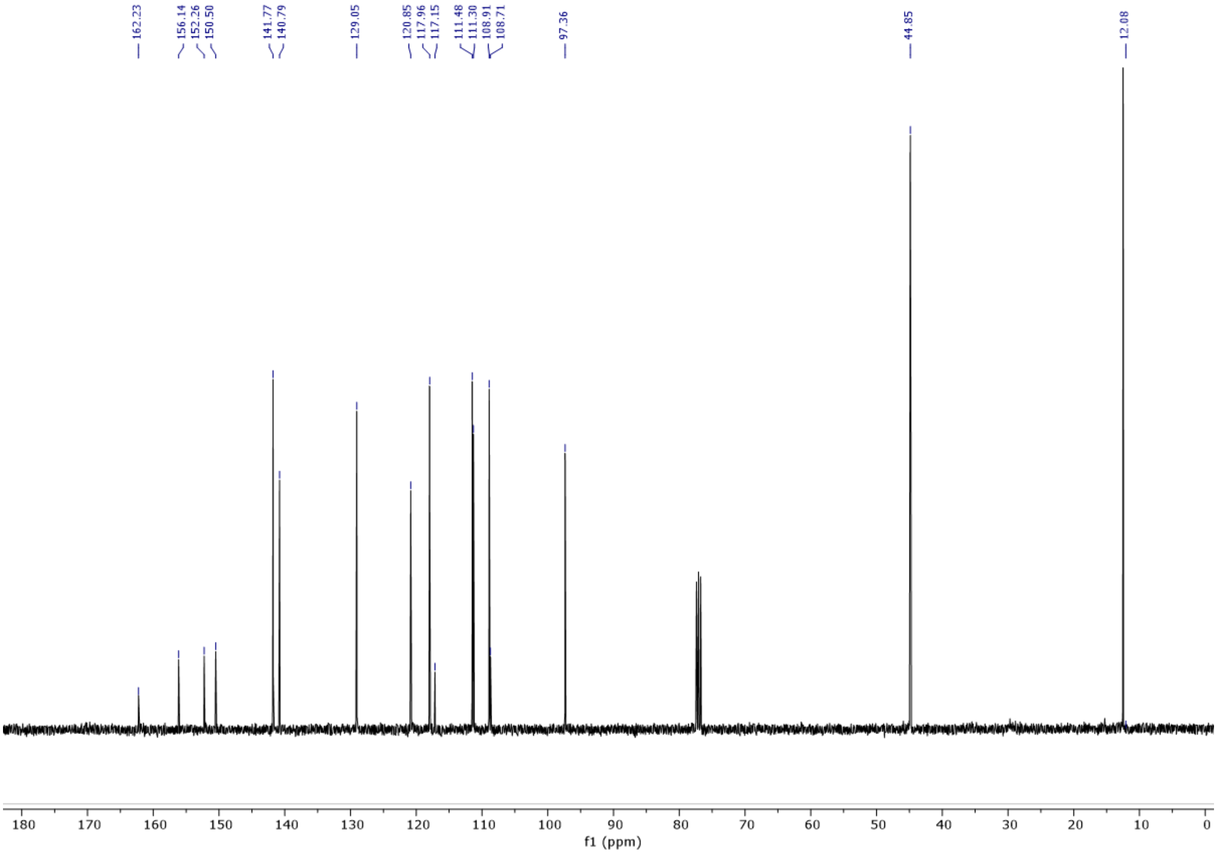

^{13}C NMR spectrum of **SC-T** (Z)

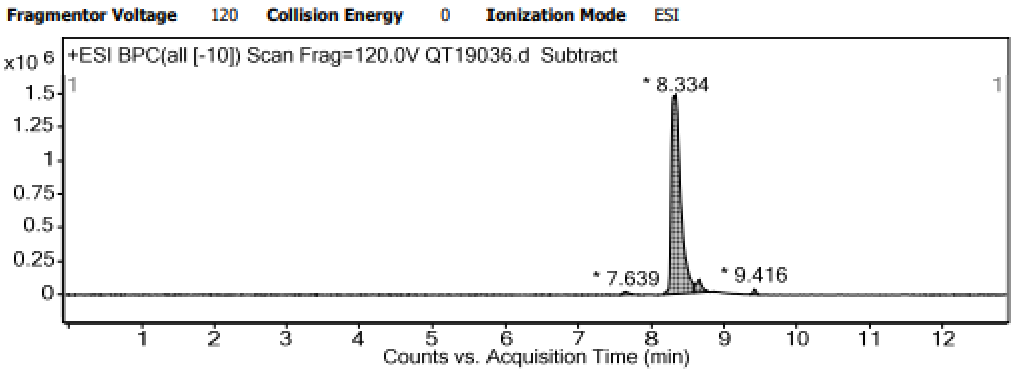

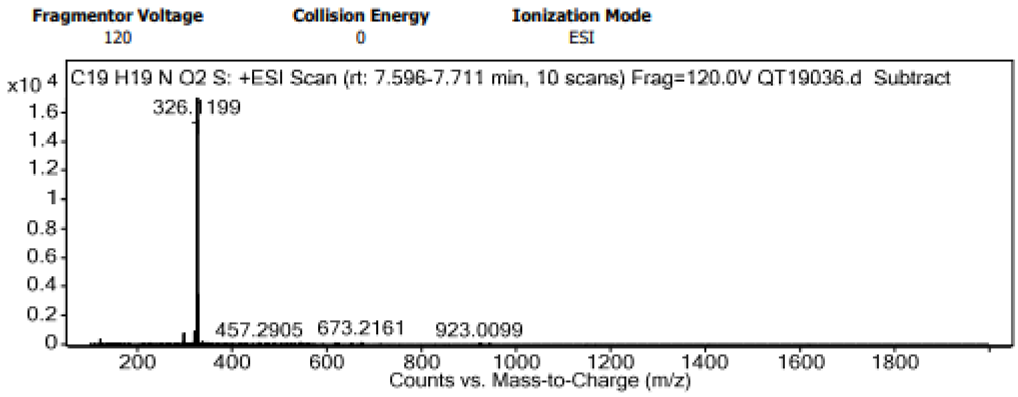

HRMS spectrum of **SC-T** (Z)

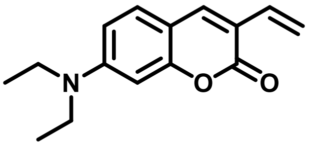

SC-V. To a solution of **4** (100 mg, 0.407 mmol, 1 eq) and Methyltriphenylphosphonium bromide (175 mg, 0.490 mmol, 1.2 eq) in THF (10 mL) was added K_2CO_3 (90 mg, 0.571 mmol, 1.6 eq). The reaction mixture was refluxed for 2 hours. The mixture was diluted with water and extracted with Et_2O. The organic phase was washed with saturated $NaHCO_3$ solution, then with brine solution, dried over $MgSO_4$, filtered and concentrated. The crude was purified by column chromatography on silica gel (DCM) to obtain **SC-V** (55 mg, 55%) as a yellowish syrup. The NMR was in accordance with the literature.[5] ^1H NMR (400 MHz, $CDCl_3$) δ 7.56 (s, 1H, ArH), 7.24 (d, J = 8.9 Hz, 1H, ArH), 6.71 – 6.61 (m, 1H, CH alkene), 6.56 (dd, J = 8.8, 2.5 Hz, 1H, ArH), 6.46 (d, J = 2.5 Hz, 1H, ArH), 6.01 (dd, J = 17.6, 1.4 Hz, 1H, CH alkene), 5.29 (dd, J = 11.3, 1.4 Hz, 1H, CH alkene), 3.39 (q, J = 7.1 Hz, 4H, CH_2 ethyl), 1.19 (t, J = 7.1 Hz, 6H, CH_3 ethyl).

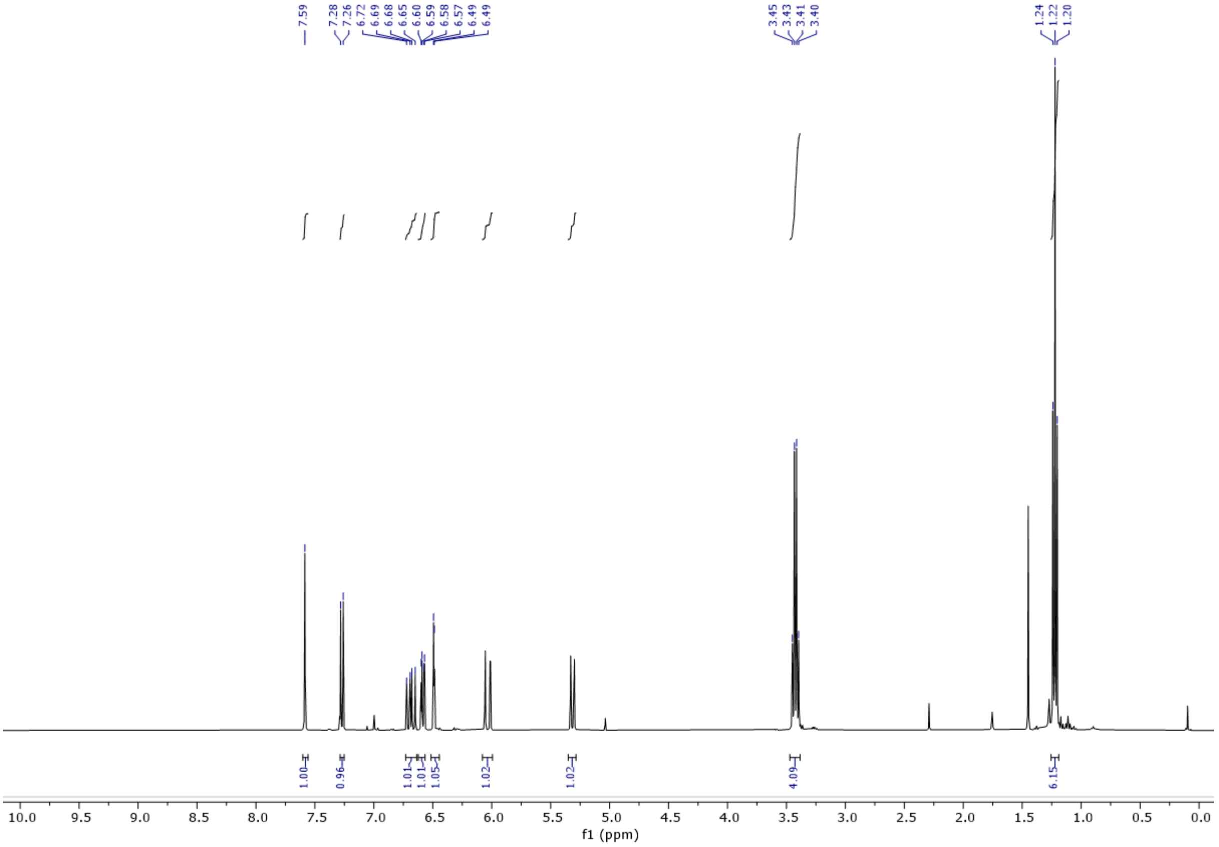

^1H NMR spectrum of **SC-V**

Synthesis of phosphonium coumarin 9

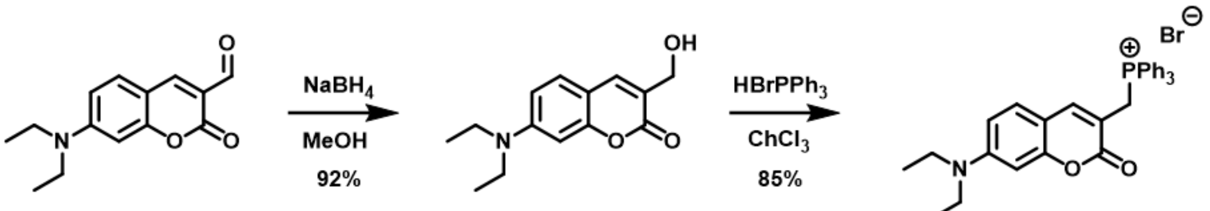

4 8 9

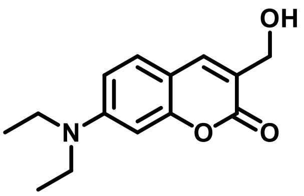

8. To a solution of **4** (500 mg, 2.04 mmol, 1 eq) at 0°C in methanol (5 mL) was added dropwise $NaBH_4$ (77 mg, 2.04 mmol, 1 eq). The solution was allowed to warm at RT. Acetone (0.1 mL) and water (5 mL) was added to the mixture. The product was extract with DCM and washed with brine, dried over $MgSO_4$. Solvents were evaporated under reduced pressure to give **8** (464 mg, 92%) as a yellow syrup. The NMR was in accordance with the literature.[6] ^{1}H NMR (400 MHz, $CDCl_3$) δ 7.55 (d, J = 0.9 Hz, 1H, ArH), 7.26 (d, J = 4.4 Hz, 1H, ArH), 6.58 (dd, J = 8.8, 2.5 Hz, 1H, ArH), 6.51 (d, J = 2.5 Hz, 1H, ArH), 4.54 (dd, J = 6.3, 0.9 Hz, 2H, CH_2OH), 3.41 (q, J = 7.1 Hz, 4H, CH_2 ethyl), 1.21 (t, J = 7.1 Hz, 6H, CH_3 ethyl).

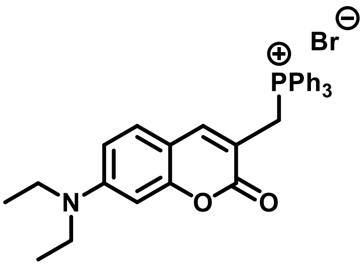

9. To a solution of **8** (320 mg, 1.29 mmol, 1 eq) in chloroform (25 ml) was added $HBrPPh_3$ (1.33 g, 3.88 mmol, 3 eq). The reaction mixture was heated at reflux overnight. The mixture was diluted with NaOH 1M (10 mL). The product was extracted with DCM dried over $MgSO_4$. The crude was purified by column chromatography on silica gel (DCM/MeOH: 98/2 to 90/10) to obtain **9** (626 mg, 85%) as a yellow foam. Rf = 0.3 (9/1 : DCM/MeOH). ^{1}H NMR (400 MHz, $CDCl_3$) δ 8.18 (d, J = 3.7 Hz, 1H, ArH), 7.93 – 7.83 (m, 6H, PPh_3), 7.79 – 7.69 (m, 3H, PPh_3), 7.62 (ddd, J = 9.1, 7.2, 3.5 Hz, 6H, PPh_3), 7.22 (dd, J = 8.9, 0.8 Hz, 1H, ArH), 6.49 (dd, J = 8.9, 2.5 Hz, 1H, ArH), 6.31 (d, J = 2.4 Hz, 1H, ArH), 5.40 (d, J = 13.8 Hz, 2H, CH_2PPh_3), 3.35 (q, J = 7.1 Hz, 4H, CH_2 ethyl), 1.16 (t, J = 7.1 Hz, 6H, CH_3 ethyl). ^{13}C NMR (101 MHz, $CDCl_3$) δ 162.40 (CO_2 Lactone), 156.37, 150.64, 147.58, 134.88, 134.85, 134.51, 134.41, 130.11, 129.98, 118.68, 117.83, 109.14, 108.43, 107.04, 96.70, 44.81 (CH_2 ethyl), 12.19 (CH_3 ethyl). RMS (ESI+) calculated for C32H31NO2P [M]+ 492.2087, found 492.2098.

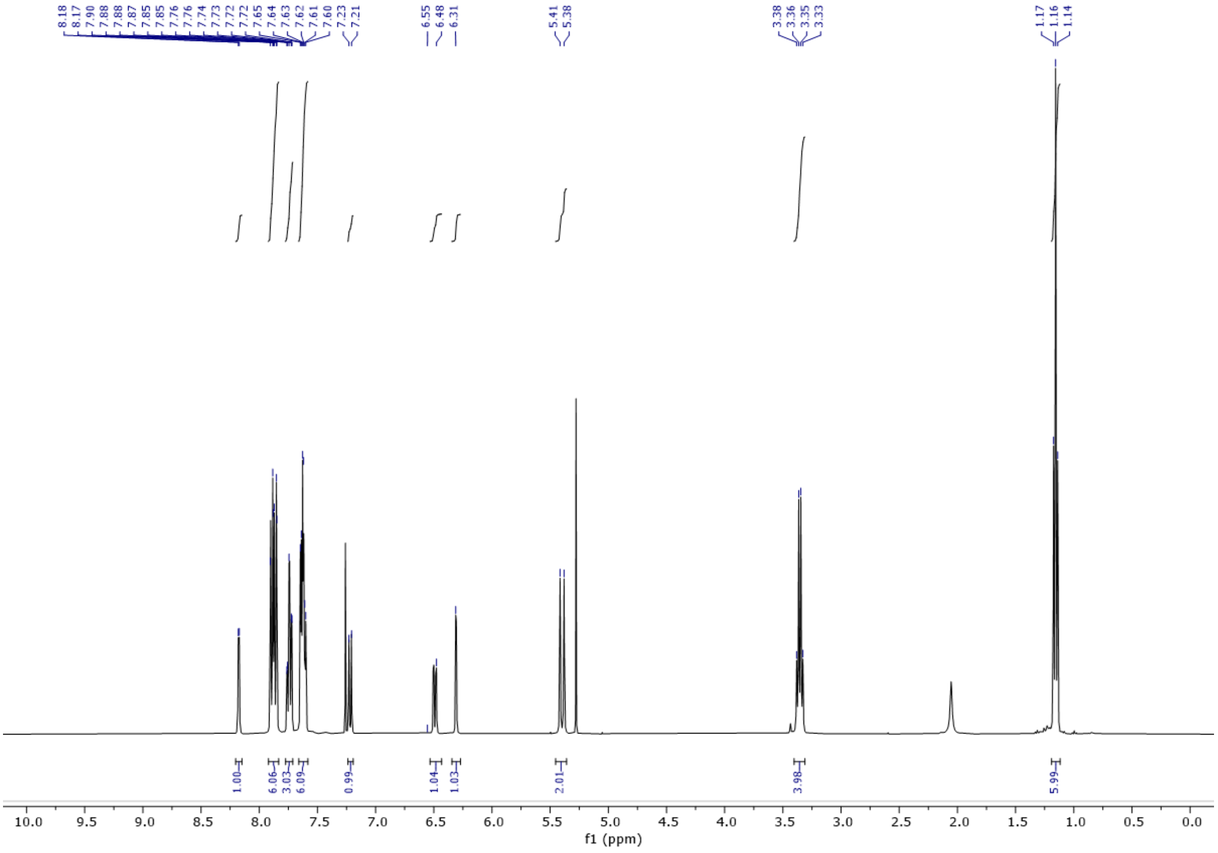

^{1}H NMR spectrum of **9**

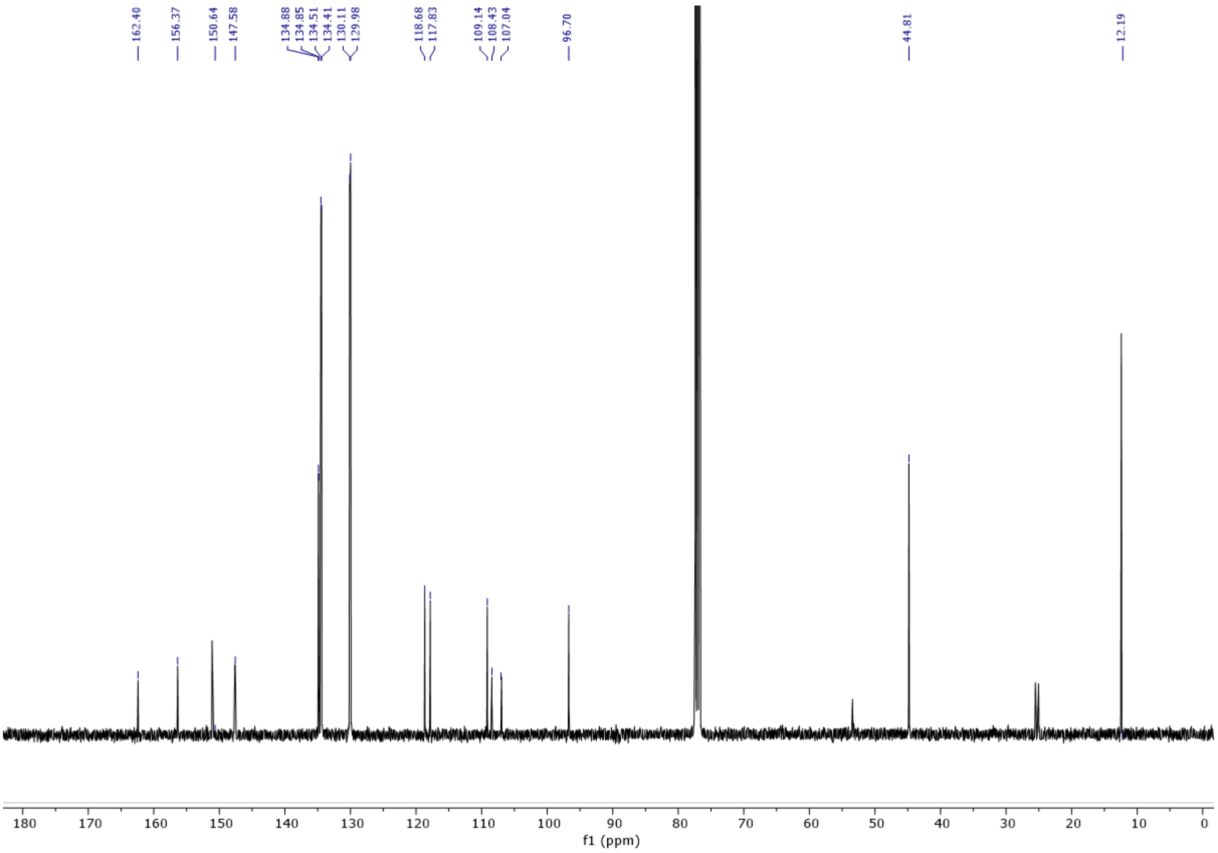

^{13}C NMR spectrum of **9**

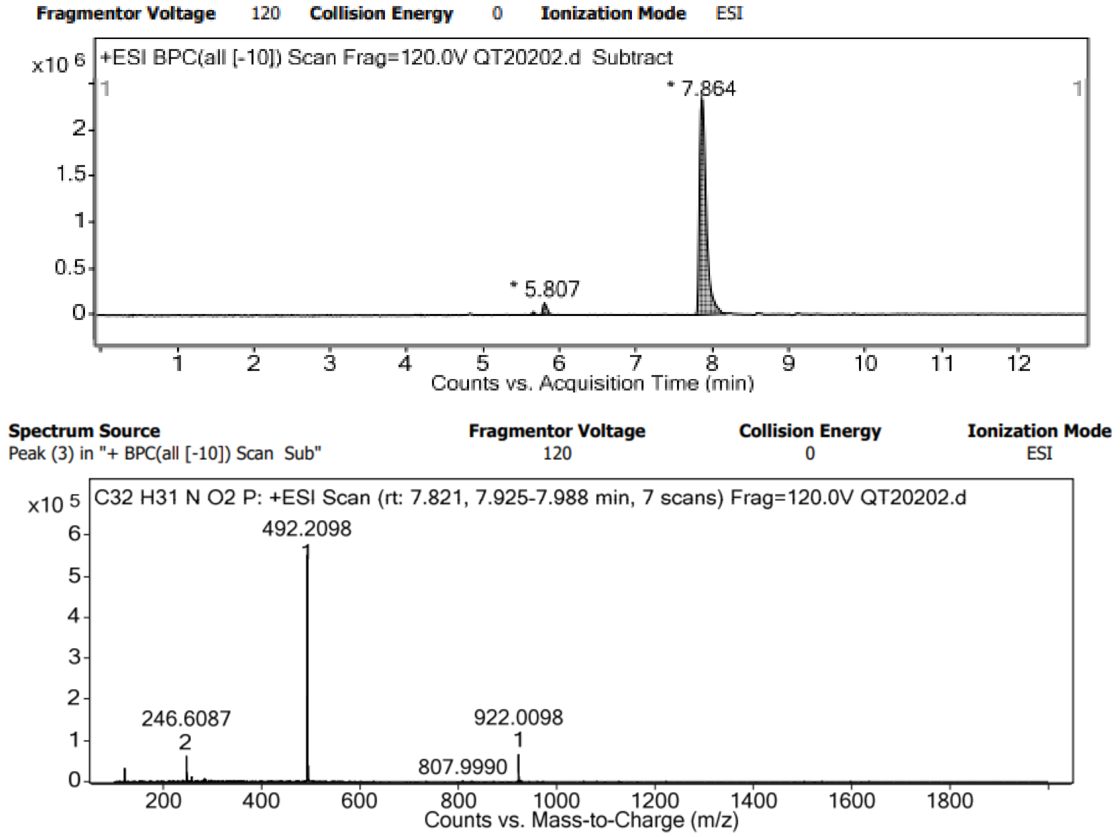

HRMS spectrum of **9**

Synthesis of SC-P via the Wittig reaction

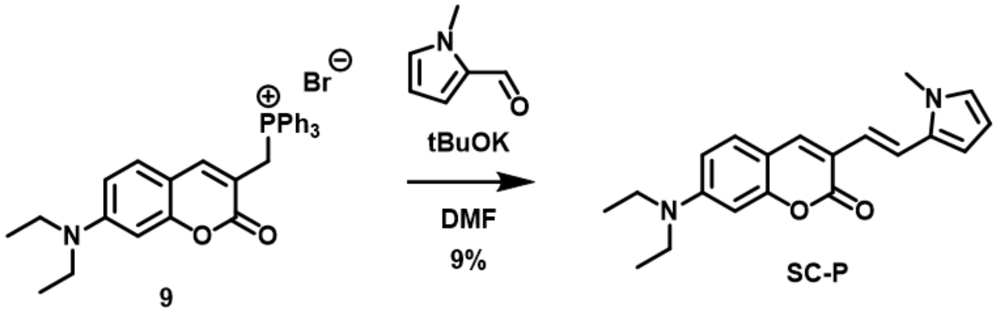

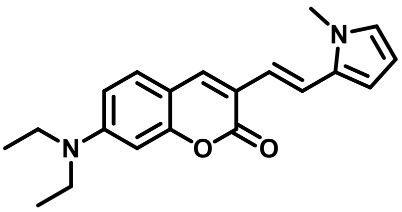

SC-P. To a solution of **9** (200 mg, 0.35 mmol, 1 eq) and 1-Methylpyrrole-2-carboxaldehyde (36 mg, 0.33 mmol, 35 µl, 0.95 eq) in DMF (10 mL) was added tBuOK (47 mg, 0.42 mmol, 1.2 eq). The reaction mixture was allowed to stir at RT for 1 hour. The mixture was diluted with LiCl 5% and extracted with Et_2O. The organic phase was dried over $MgSO_4$, filtered and concentrated. The crude was purified by column chromatography (Heptan/DCM/EtOAc : 5/4/1) to obtain **SC-P** (10 mg, 9%) as an orange amorphous solid. Rf = 0.47 (Heptan/DCM/EtOAc : 5/4/1) . ^{1}H NMR (400 MHz, $CDCl_3$) δ 7.54 (d, J = 15.5 Hz, 2H, CH alkene and ArH), 7.26 (d, J = 8 Hz, 1H, ArH), 6.77 (dd, J = 16.0, 0.7 Hz, 1H, CH alkene), 6.63 (dd, J = 2.6, 1.7 Hz, 1H, H pyrrole), 6.58 (dd, J = 8.8, 2.5 Hz, 1H, ArH), 6.51 (d, J = 2.5 Hz, 1H, ArH), 6.49 (ddd, J = 3.8, 1.7, 0.6 Hz, 1H, H pyrrole), 6.15 (ddd, J = 3.6, 2.6, 0.6 Hz, 1H, H pyrrole), 3.71 (s, 3H,N-methyl), 3.42 (q, J = 7.1 Hz, 4H, CH_2 ethyl), 1.22 (t, J = 7.1 Hz, 6H, CH_3 ethyl). ^{13}C NMR (101 MHz, $CDCl_3$) δ 161.25 (CO_2 Lactone), 155.16, 150.06, 137.33, 132.67, 128.40, 123.73, 120.66, 119.24, 118.38, 109.34, 109.04, 108.39, 106.50, 97.22, 45.80 (CH_2 ethyl), 34.88

(CH$_3$ N-mehtyl), 12.52 (CH$_3$ ethyl). HRMS (ESI+) calculated for $C_{20}H_{22}N_2O_2$ [M+H]$^+$ 323.1715, found 323.1766.

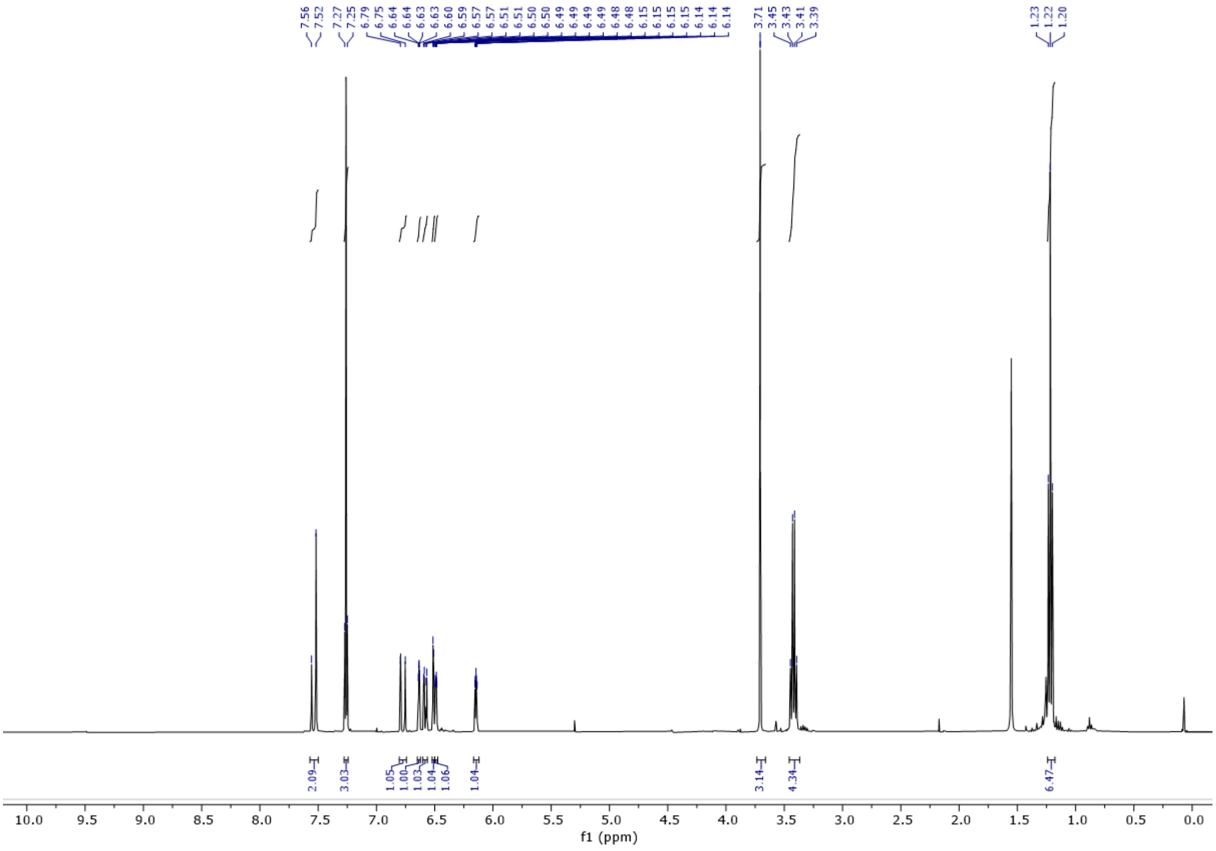

^{1}H NMR spectrum of **SC-P**

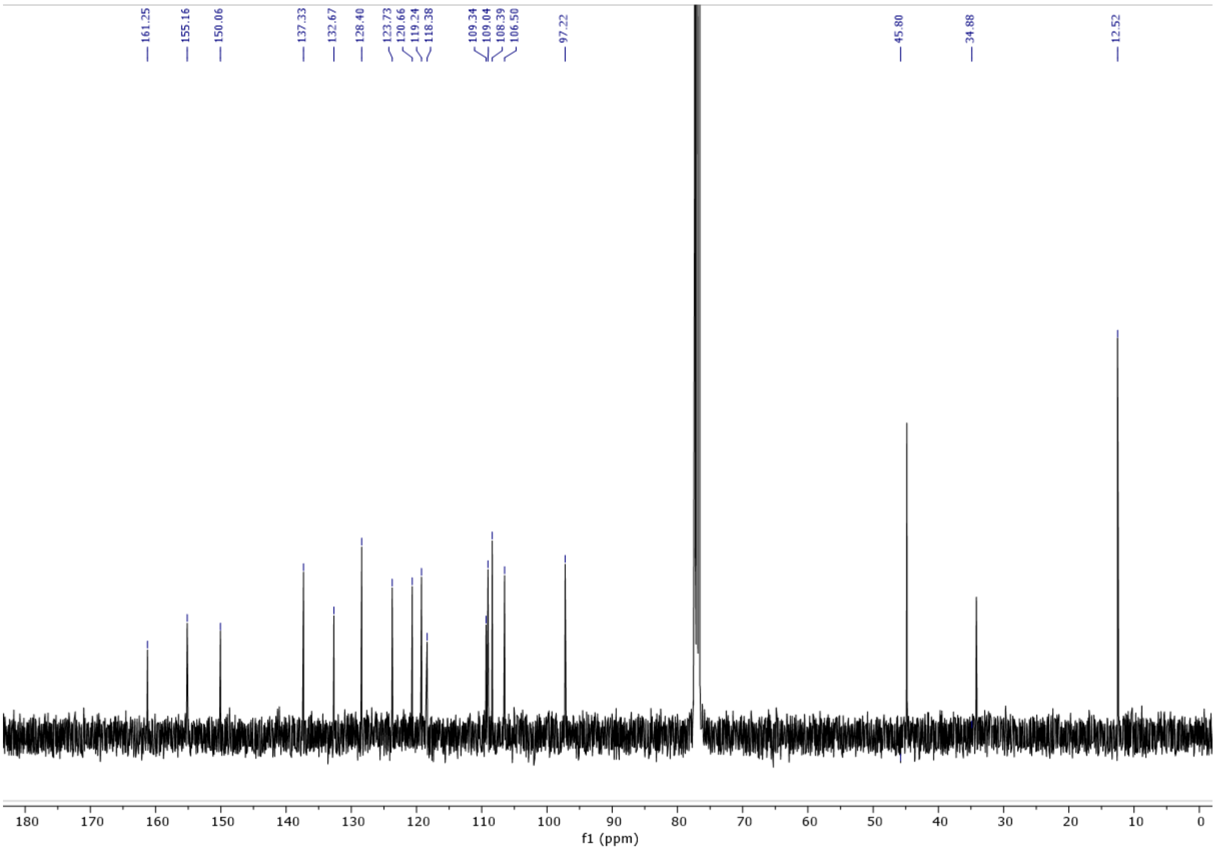

^{13}C NMR spectrum of **SC-P**

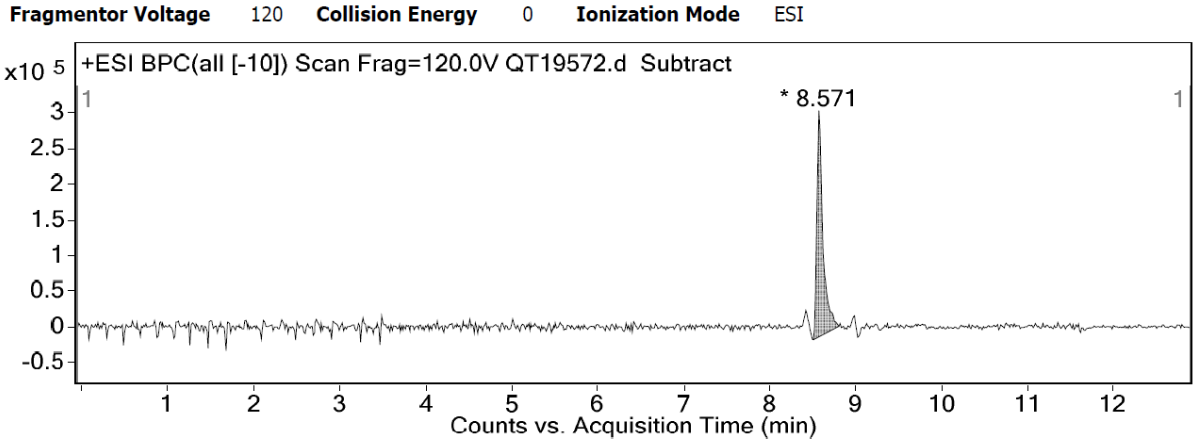

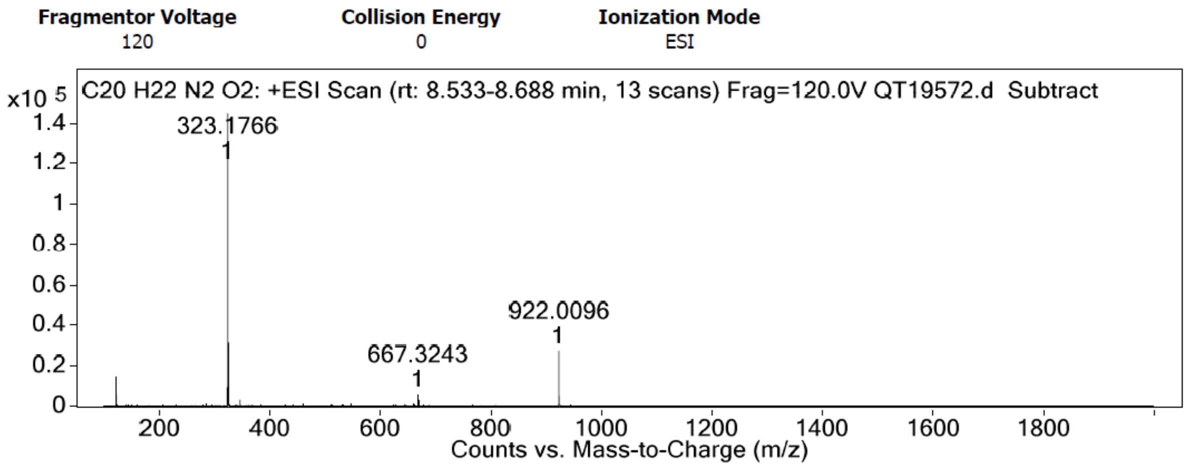

HRMS spectrum of **SC-P**

2) **Materials and methods**

All starting materials for synthesis were purchased from Alfa Aesar, Sigma-Aldrich, or TCI Europe and used as received unless stated otherwise. NMR spectra were recorded on a Bruker Avance III 400 MHz spectrometer. Data are presented as follows: chemical shift (ppm), multiplicity (s = singlet, d = doublet, t = triplet, q = quartet, quint = quintet, dd = doublet of doublets, m = multiplet, br = broad), coupling constant J (Hz) and integration. Mass spectra were obtained using an Agilent Q-TOF 6520 mass spectrometer. The presented data were obtained on the E isomers of the SCs. Absorption spectra were recorded on a Cary 4000 spectrophotometer (Varian). Fluorescence spectra were recorded on a Fluoromax-4 (Jobin Yvon, Horiba) spectrofluorometer. Emission measurements were systematically done at 20˚C, unless indicated otherwise. All the spectra were corrected from the wavelength-dependent response of the detector. HPLC analyses were performed on Agilent 1260 Infinity II system equipped with Interchim PF5C18AQ-250/046 reversed phase column with gradual eluting from water to acetonitrile for 20 min at 1.5 ml/min. cLogP value of **SC-P** was determined using the software Chemdraw (Perkin Elmer).

The fluorescence quantum yields φ_F were determined following the following equation:

$$\varphi_F = \varphi_{ref} \times \frac{I_{fluo}^{sample}\, d\lambda}{I_{fluo}^{ref} d\lambda} \times \frac{OD_{ref}}{OD_{sample}} \times \frac{n_{sample}^2}{n_{ref}^2}$$

with OD optical density at the excitation wavelength, and n refraction index of the solvent. 7-(diethylamino)coumarin-3-carboxylic acid (φ_F = 0.93 in dioxane[7]) was used as reference.

Photoconverison studies. Laser spectroscopy and conversion were performed using 3 × 3 mm optical path length quartz cuvettes of 45 µL. Excitation was provided by a cw laser diode (488 and 405 nm, Oxxius, Lannion, France) and photons were detected by a QE pro spectrometer from Ocean Optics. All measurements were performed at room temperature. The kinetic rate of phototransformation, k_{Pt}, was determined by fitting the emission decrease (integrated spectra) of the photo-converting dye over time as described in Moerner's method[8] and according to the following equation:

$$A(t) = A_\alpha e^{(-k_\alpha t)} + A_\beta e^{(-k_\beta t)}$$

Where A(t) is the emission signal over time of the photo-converting dye.

The kinetic rate of phototransformation (k_{Pt}) is given as an average of the two kinetic constants of the two-exponential fit:

$$k_{Pt} = \frac{\frac{A_\alpha}{k_\alpha} + \frac{A_\beta}{k_\beta}}{\frac{A_\alpha}{k_\alpha{}^2} + \frac{A_\beta}{k_\beta{}^2}}$$

Then the quantum yield of phototransformation ϕ_{Pt} is given by:

$$\phi_{Pt} = \frac{k_{Pt} N_a}{I \cdot 2303 \cdot \varepsilon}$$

Where I is the irradiance at the sample ($W.cm^{-2}$), ε the molar-absorption coefficient of the dye at the excitation wavelength ($L.mol^{-1}.cm^{-1}$) and N_a the Avogadro's constant (mol^{-1}).

Irradiance is defined as:

$$I = \frac{P\lambda}{Shc}$$

Where S is the surface irradiated (S = 0.15 cm²), h the Planck's constant, c is the speed of light (m.s^{-1}), P the power of the laser (in W) and λ the wavelength of the laser used for conversion (m).

Based on our model, we hypothesized that **cSC** was similar to **SC-V**. The chemical yield (η) is obtained by comparing the fluorescence intensity of **SC** before conversion (Fl_A) and after conversion (Fl_B) knowing Irradiance of the sample (I) and the Brightness (B) defined as ε x φ_F at the laser's wavelength of each dye:

$$\eta = \frac{Fl_B \cdot B_A \cdot I_A}{Fl_A \cdot B_B \cdot I_B}$$

Finally, the quantum yield of photoconversion (φ_{Pc}) was obtained by multiplying the quantum yield of phototransformation by the chemical yield.

$$\phi_{Pc} = \phi_{Pt}\, \eta$$

$$\phi_{Pt} = \phi_{Bl} + \phi_{Pc}$$

Consequently, when the dye is not convertible $\phi_{Pt} = \phi_{Bl}$

The photobleaching of the converted forms (**cSC**) was performed at 405 nm (237 mW.cm^{-2}) (after the photoconversion step) and the quantum yield of photobleaching was determined as described above.

An attempt to obtain the quantum yield of $^1O_2$ generation (φ_Δ) of **SC-P** was performed by monitoring the absorbance change of a solution of 1,3-Diphenylisobenzofuran (DPBF) at 100 µM and **SC** at 5 µM over time and upon laser irradiation. Unfortunately, the absorbance of **SC-P** decreases during the experiment showing the competitive reactivity of **SC-P** and the DPBF sensor towards $^1O_2$, thus leading to unusable results.

Cell Culture. Hela cells were grown on poly-D-ornithine (Sigma Aldrich, P8638) coated #1.5 18 mm glass coverslips in DMEM (4.5 g/L^{-1} D-Glucose) supplemented with 10% fetal bovine solution (Gibco™) 1% antibiotic solution (penicillin Streptomycin) and 1% Glutamax. They were incubated in humidified incubator with 5% CO_2. Astrocytes were prepared from embryonic day 18 (E18) Sprague Dawley rat embryos. Brains were extracted from the embryos and hippocampi were dissociated in 0,25% tryspin solution at 37°C for 15 min and then seeded in MEM medium (Gibco™) supplemented with 10% horse serum, 1% Glutamax, 3% Glucose (0.2 g.L^{-1}) and 1% antibiotic at 37 °C at an approximate humidity of 95–98% with 5% CO_2. 25 × 103 cells were seeded in 12 well plates on poly-D-ornithine (Sigma Aldrich, P8638) coated #1.5 (18 mm) glass coverslips (Menzel-Gläser, Thermo Scientific). After 4 h the medium was removed and replaced with the same initial medium. Cultures were kept at 37 °C for up to 14 days.

Cellular imaging. Conversion and confocal imaging have been performed on a Zeiss LSM880 – ElyraPS1 microscope equipped with a 63x (1.4 NA)/ Oil immersion objective. **SC-P** was excited with a 488 nm laser line and emission was collected between 496 and 602 nm. **cSC-P** was excited with a 405 nm laser line and emission was collected between 407 and 448 nm. SMCy5.5 or Membright Cy5.5 were excited with a 633 nm laser line and emission was collected between 640 and 750 nm. 488 nm laser line

is from an argon laser (458, 475, 488, 496, 514 nm) and 405 nm and 633 nm wavelengths were from a laser diode and a Helium-Neon laser.

Cells were incubated with **SC-P**, SMCy5.5 or Membright Cy5.5 in Krebs-Ringer solution at pH 7.42 at 37°C. **SC-P** was used at 5 µM, SM Cy5.5 at 0.2 µM and Membright Cy5.5 was used at 0.05 µM. We used a mixture containing all the probes (**SC-P** and SMCy5.5 or **SC-P** and Membright Cy5.5). Cells were incubated for 2 hours with the labelling mixture, washed three times in a row with Krebs-Ringer solution pre-warmed at 37°C and then imaged in Krebs-Ringer solution during imaging.

Acquisition were done with Zen Black software. Image size were fixed using ideal sampling mode. In order to follow the dynamic intensity over time, images were neither averaged in line nor in frame to speed up the process. Imaging was performed using low laser power: between 0.5% for Hela and 2% for astrocytes with both 488 nm and 405 nm laser line, 2% of 633 nm laser line for SMCy5.5 and 15% for MemBright. To convert **SC-P** into **cSC-P**, we used the FRAP module on Zen Black software: dwell time during conversion were twice shorter than during image acquisition and acquired 5 times before acquisition. Conversion was done between frame 1 and 2.

Photoconversion in cells. Imaging was done using 0.5 percent of 488 nm laser line, and conversion was triggered in dedicated region of interest using 3 repeated scans at low speed using 100 percent 488 nm laser line. Non-converted form and converted form were then image at fastest rate using sequential acquisition with 0.5% of 488 nm laser line (for non-converted form) and 0.3% of 405 laser line for converted form for each time point.

Conversion yield in cells. To evaluate conversion yield we performed lipid droplet segmentation using Wavelet Icy spot detector plugin in both 405 and 488 channels. We identify spots in both channels before and after conversion. The yield of conversion was calculated as the proportion of 488 Lipid droplets spots evaluated just before stimulation that give rise to 405 positive lipid droplets in the following frame after conversion. Over 3 independent stimulated cells we evaluated the conversion yield as 91.6 ± 8.0 %. We also evaluate the proportion of 488 spots that totally disappeared from 488 channel after conversion as 80.5 %, the remaining 19.5% being the brightest lipid droplets that were only partially converted and that had residual 488 signal.

Image Quantifications. For each condition, batch of images used for quantification (Intensity profile, Colocalization analysis and tracking analysis) were analysed using the same parameters of segmentation. Image analysis was done on Icy software (Icy spot detector plugin).[9]

Intensity profile. Intensity profile of **SC-P** and **cSC-P** were done with Icy plugin ROI Intensity Evolution. ROIs were determined by fusing **SC-P** and **cSC-P** mask using Spot Detector plugin and merge function. Intensity were then tracked over time.

Colocalization analysis. In order to determinate the percentage of association of **SC-P** and **cSC-P** with SMCy5,5 we used Icy SODA plugin.[10] First, we used Spot Detector plugin to detect **SC-P**, **cSC-P** and SMCy5.5. Spots detection of **SC-P** and SMCy5,5, **SC-P** and **cSC-P** or **SC-P** and **cSC-P** were then used in the SODA plugin to obtain the percentage of association whenever they are associated.

Tracking analysis. Spot tracking of **SC-P**, **cSC-P** and SMCy5.5 were done using Icy spot tracking plugin, showing that they indeed follow the same trajectory with the same speed. To track **SC-P**, **cSC-P** and SMCy5.5 we use the spot detector and then spot tracking plugin in Icy (with parameter for target motion re-estimated online). The data were exported in excel and the quantification was performed in PRISM software. Average speeds were compared using ANOVA (with Dunn's correction).

3) **Spectroscopic studies**

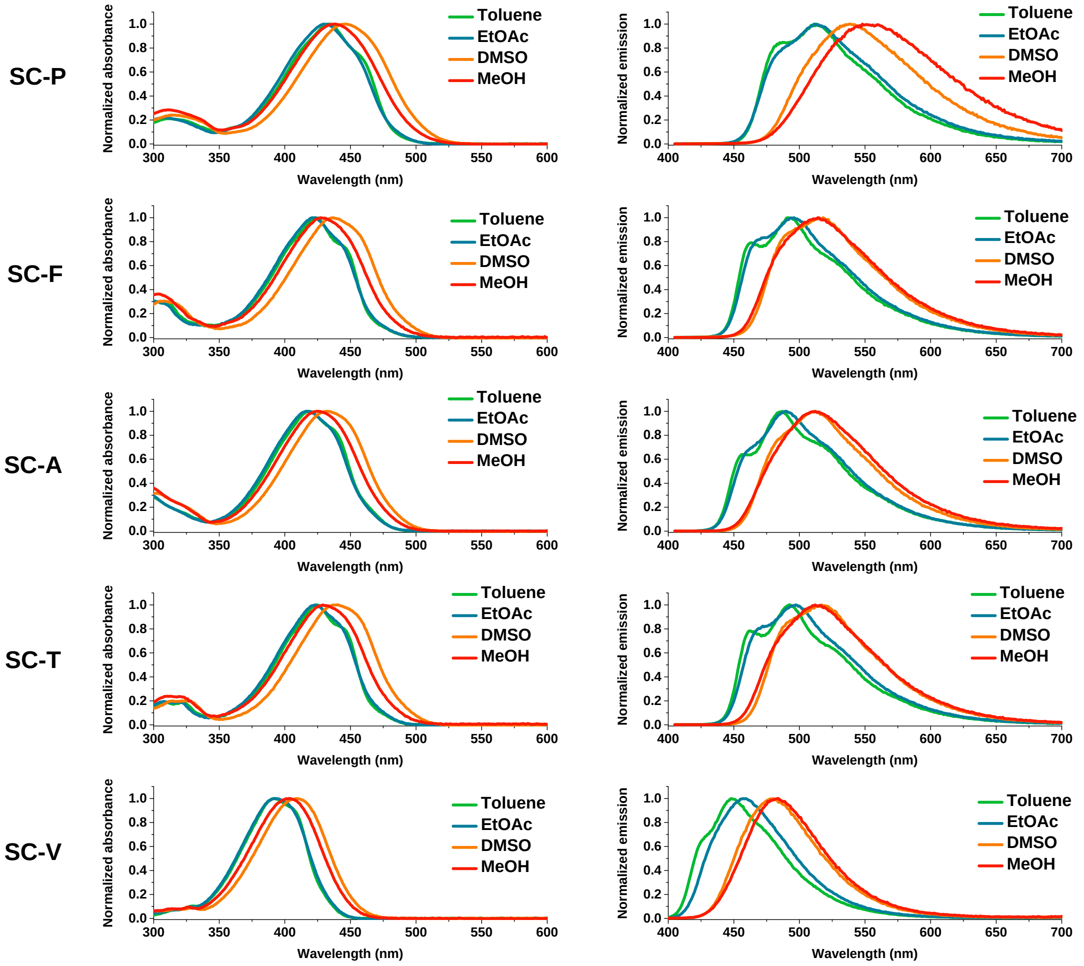

Figure S1. Absorption (left) and emission (right) spectra of styryl coumarins **SCs** in various solvents. Excitation wavelength was 400 nm for **SC-P**, **SC-F**, **SC-A**, **SC-T** and 370 nm for **SC-V**. The concentration of the probes was 5 μM.

Table S1. Photophysical properties of **SCs** in various solvents.

SC-P	λ_{Abs} (nm)	ε ($M^{-1}.cm^{-1}$)	λ_{Em} (nm)	φ_F	B[a] ($M^{-1}.cm^{-1}$)
**Toluene**	434	56,200	511	75	42,100
**EtOAc**	430	55,600	512	68	37,800
**DMSO**	445	51,000	539	86	43,900
**MeOH**	438	49,000	551	49	24,000

SC-F	λ_{Abs} (nm)	ε ($M^{-1}.cm^{-1}$)	λ_{Em} (nm)	φ_F	B[a] ($M^{-1}.cm^{-1}$)
**Toluene**	423	49,400	491	74	36,500
**EtOAc**	421	46,800	496	72	33,700
**DMSO**	437	45,000	518	88	39,600
**MeOH**	428	40,000	514	72	28,800

SC-T	λ_{Abs} (nm)	ε ($M^{-1}.cm^{-1}$)	λ_{Em} (nm)	φ_F	B[a] ($M^{-1}.cm^{-1}$)
**Toluene**	425	54,400	492	64	34,800
**EtOAc**	423	51,800	497	62	32,100
**DMSO**	439	47,400	517	87	41,200
**MeOH**	429	46,400	512	65	30,200

SC-A	λ_{Abs} (nm)	ε ($M^{-1}.cm^{-1}$)	λ_{Em} (nm)	φ_F	B[a] ($M^{-1}.cm^{-1}$)
**Toluene**	418	44,800	487	80	35,800
**EtOAc**	418	43,700	490	77	33,600
**DMSO**	432	44,780	512	89	39,900
**MeOH**	425	41,960	512	77	32,300

SC-V	λ_{Abs} (nm)	ε ($M^{-1}.cm^{-1}$)	λ_{Em} (nm)	φ_F	B[a] ($M^{-1}.cm^{-1}$)
**Toluene**	392	30,260	448	82	24,800
**EtOAc**	393	27,580	457	79	21,800
**DMSO**	410	29,820	480	87	25,900
**MeOH**	403	24,540	483	79	19,400

[a] B is the brightness (ε × φ_F)

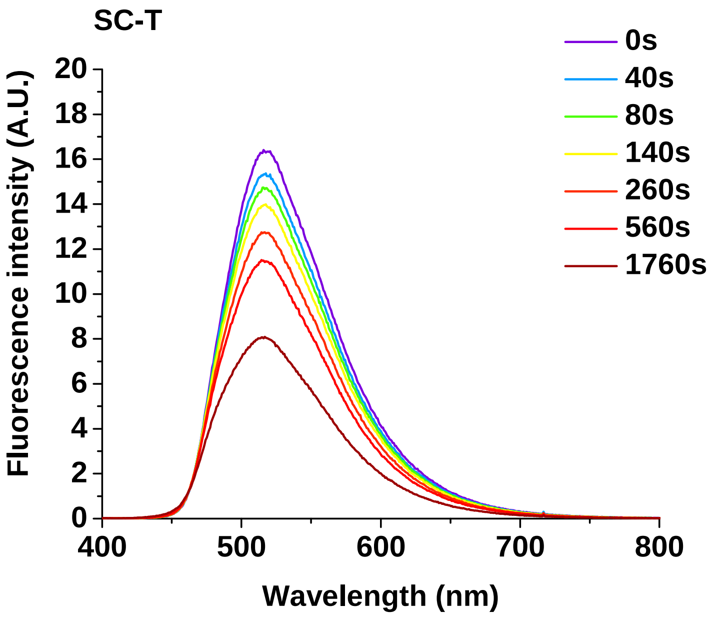

Figure S2. Emission spectra of **SC-T** upon laser irradiation at 488 nm (33 $mW.cm^{-2}$). Spectra were acquired with a 405 nm laser (4 $mW.cm^{-2}$).

4) **Analysis of photoproducts**

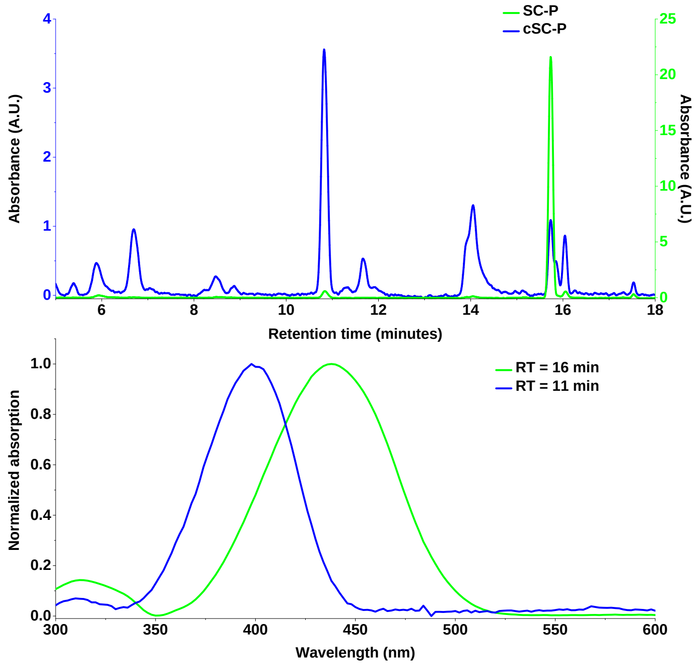

Figure S3. Analysis of the photoproduct of SC-P. (A) HPLC trace of **SC-P** before and after irradiation at 488 nm. HPLC was performed on a C_{18} column with an acetonitrile/water gradient 0/100 to 100/00 in 20 min at 1.5 ml/min the signal was detected at 405 nm. (B) Absorption spectra of **SC-P** and of one of its photoproducts (**cSC-P**).

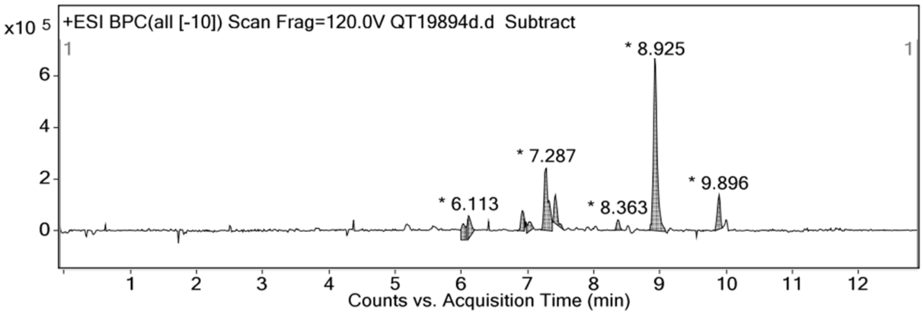

Retention time	M/Z	[M] = 322
**6.0 min**	**355.1653**	**[M+33]**
**6.1 min**	**355.1656**	**[M+33]**
6.9 min	360.1448	[M+38]
7.0 min	373.1757	[M+51]
**7.3 min**	**355.1664**	**[M+33]**
**7.4 min**	**355.1660**	**[M+33]**
8.3 min	347.1689	[M+25]
8.4 min	692.3200	[M+370]
8.9 min	338.1646	[M+16]

Proposed structures

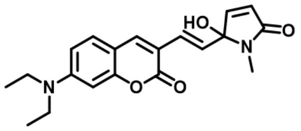

Calculated [M+32+H]$^+$: 355.1658

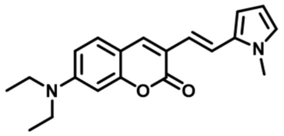

Calculated [M+H]$^+$: 323.1760

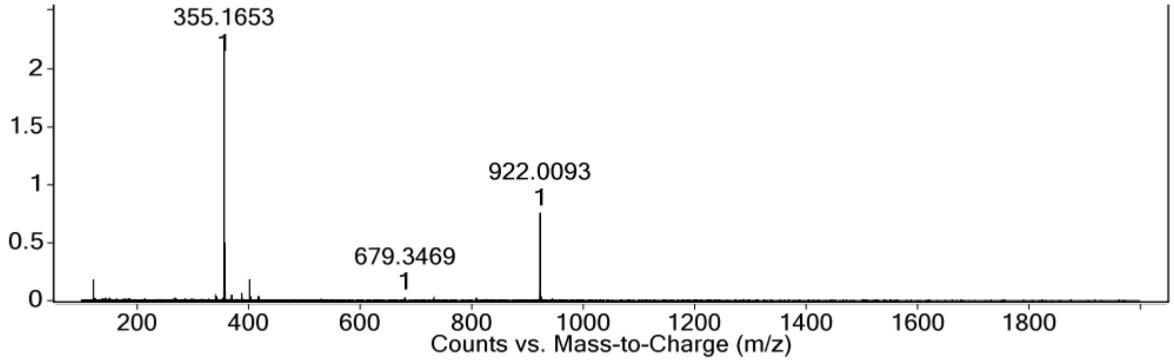

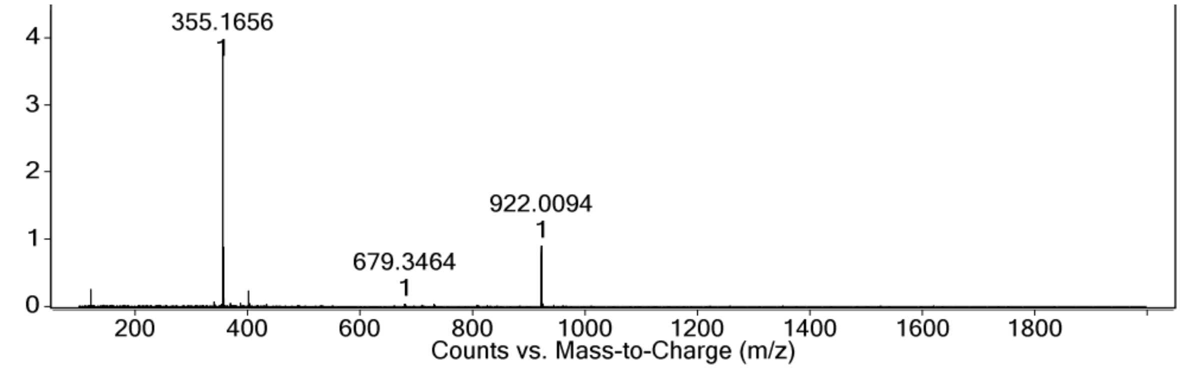

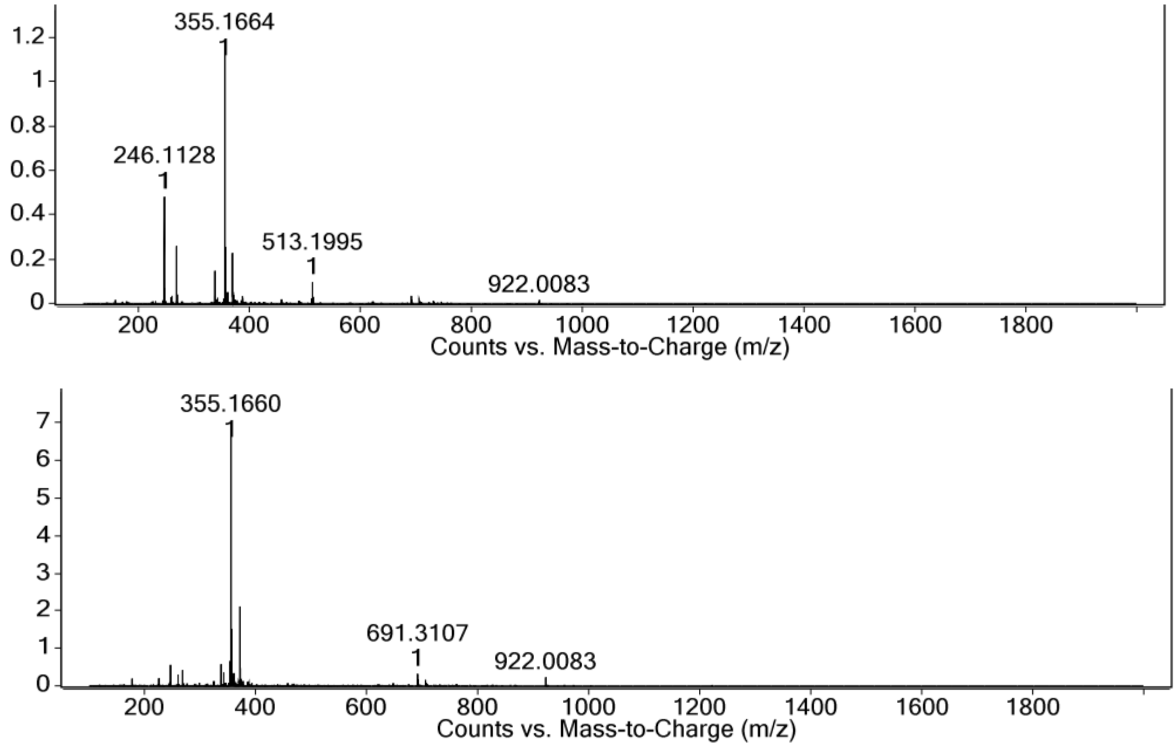

Figure S4. HPLC mass analysis of **SC-P** photoproducts (**cSC-P**) arising from reaction with singlet oxygen M+O_2+H [M+33] and some corresponding HRMS spectra.

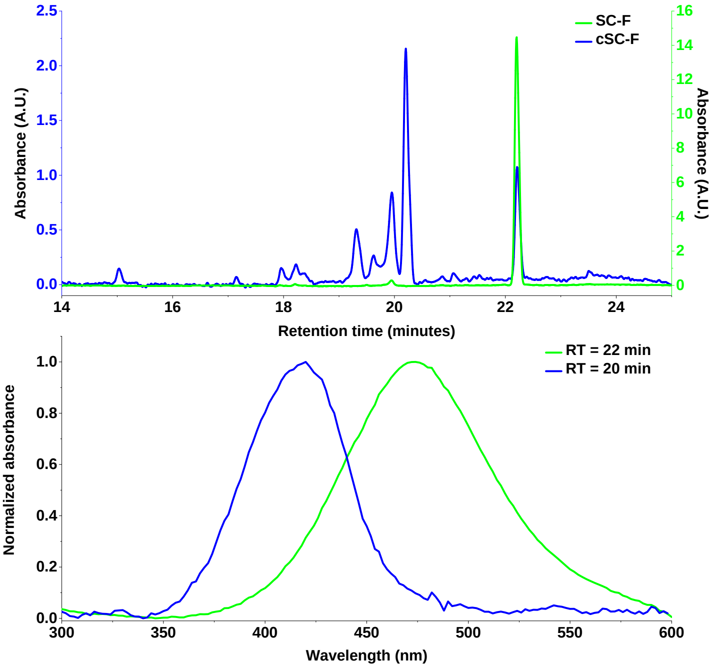

Figure S5. Analysis of the photoproduct of SC-F. (A) HPLC trace of **SC-F** before and after irradiation at 488 nm. HPLC was performed on a C_{18} column with an acetonitrile/water gradient 0/100 to 100/00 in 20 min then 100/0 in 5 min at 1.5 ml/min. The signal was detected at 405 nm. (B) Absorption spectra of **SC-F** and of one of its photoproducts (**cSC-F**).

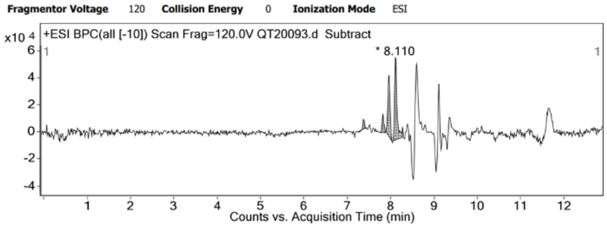

Retention time	M/Z	[M] = 309
7.3 min	342.1340	[M+33]
7.8 min	342.1346	[M+33]
8.0 min	326.1388	[M+17]
8.2 min	356.1496	[M+47]

Proposed structures

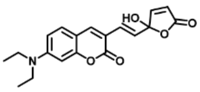

Calculated $[M+32+H]^+$: 342.1341

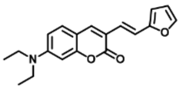

Calculated $[M+H]^+$: 310.1443

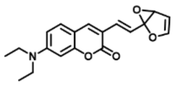

Calculated $[M+16+H]^+$: 326.1392

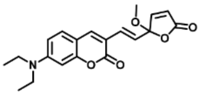

Calculated $[M+46+H]^+$: 356.1498

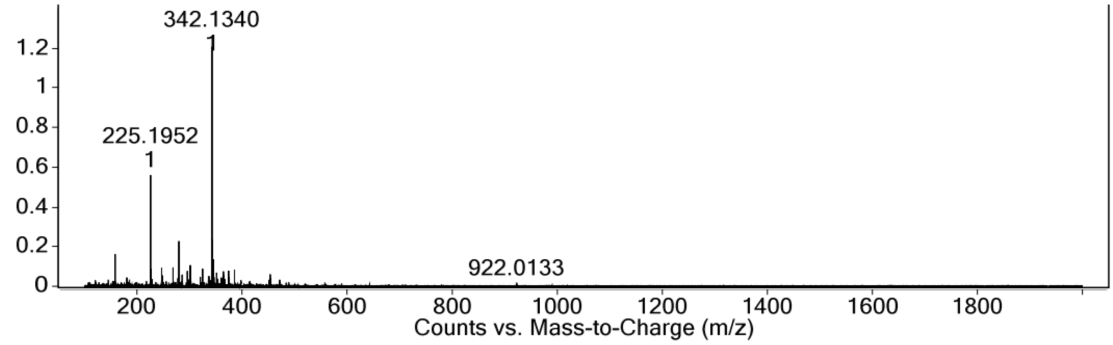

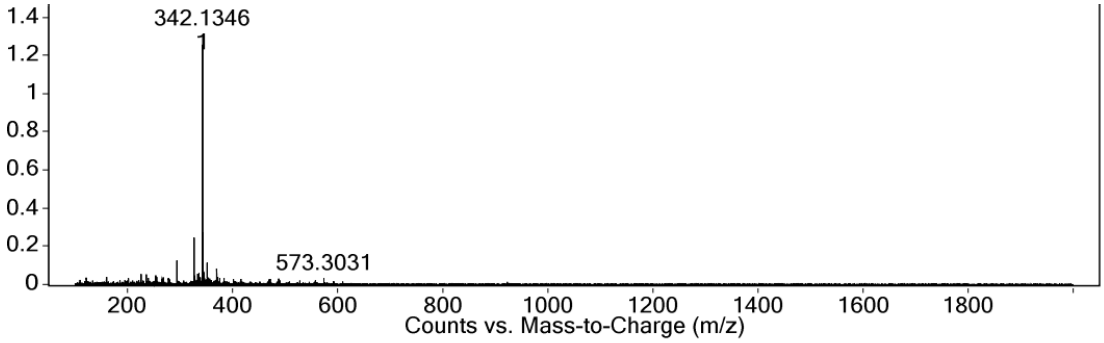

Figure S6. HPLC mass analysis of **SC-F** photoproducts (**cSC-F**) arising from reaction with singlet oxygen $M+O_2+H$ [M+33] and some corresponding HRMS spectra.

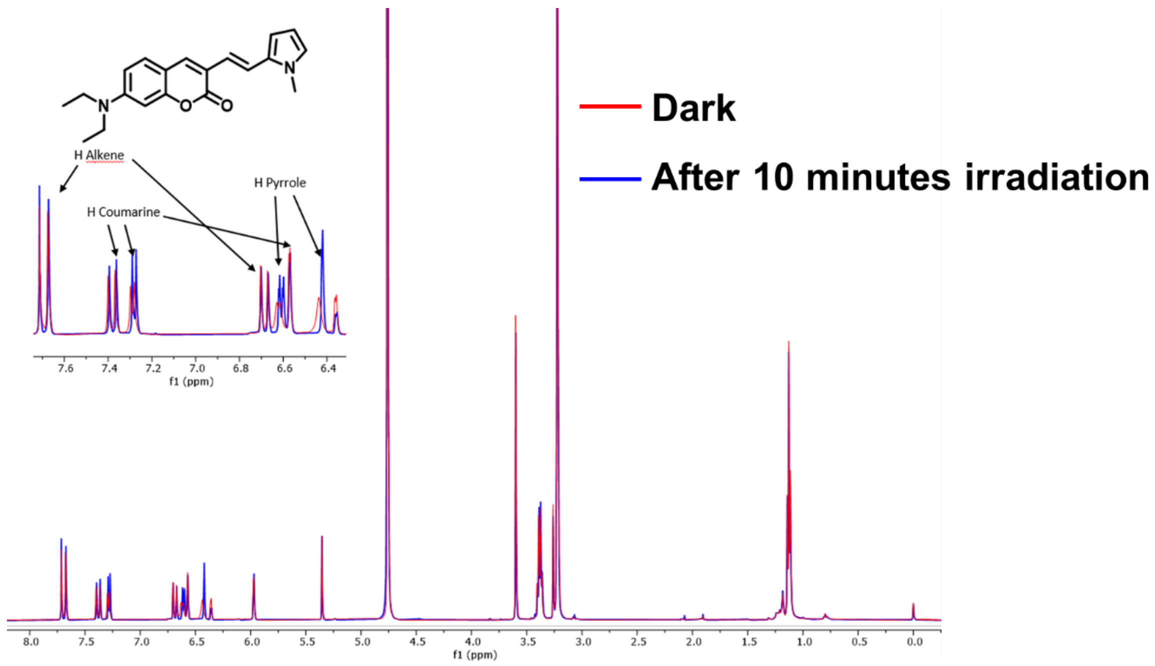

Figure S7. ^{1}H NMR spectra of SC-P in MeOD (15.5 mM) before (red line) and after irradiation (blue line). The irradiation was performed using a 488 nm laser (33 mW/cm²) for 10 minutes.

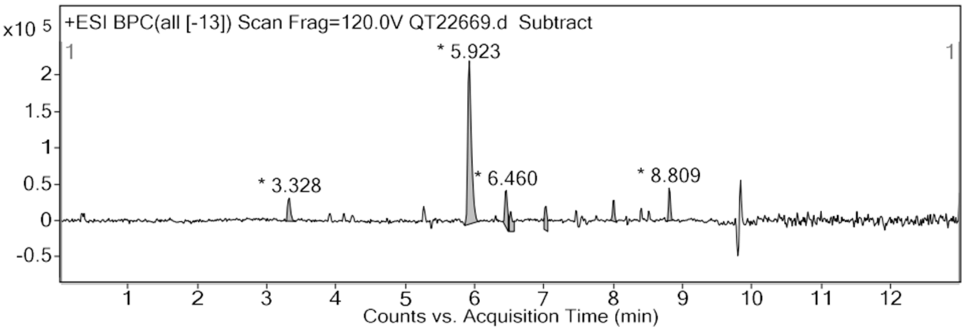

Retention time	M/Z	[M] = 349
3.3 min	391.2875	[M+42]
5.9 min	246.1130	[M-103]
6.4 min	380.1497	[M+31]
6.5 min	252.1229	[M-97]
7.0 min	251.1640	[M-98]
**8.0 min**	**350.1755**	**[M+1]**
8.8 min	637.3049	[M+288]

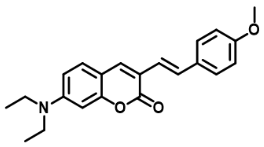

Calculated [M+H]⁺ : 350.1756

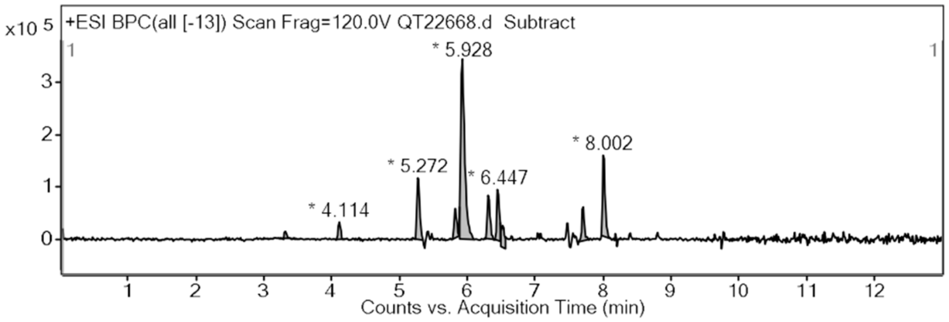

Retention time	M/Z	[M] = 325
3.3 min	433.3289	[M+108]
4.1 min	445.2314	[M+120]
5.2 min	280.1185	[M-45]
5.8 min	278.1390	[M-47]
5.9 min	246.1134	[M-79]
6.3 min	194.1175	[M-131]
6.4 min	390.1377	[M+65]
6.5 min	252.1229	[M-73]
7.6 min	358.1479	[M+33]
**8.0 min**	**326.1215**	**[M+1]**

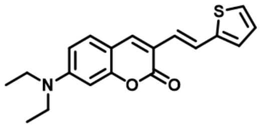

Calculated [M+H]+ : 326.1215

Figure S8. Analysis of SC-A and SC-T photoproducts. (A) HPLC trace of **SC-A** (top) and **SC-T** (bottom) after photobleaching at 488 nm. The correspond mass of the peaks are noted in the table showing oxidized [M+nO_2] and cleaved photoproducts (lower mass compared to the starting **SC**).

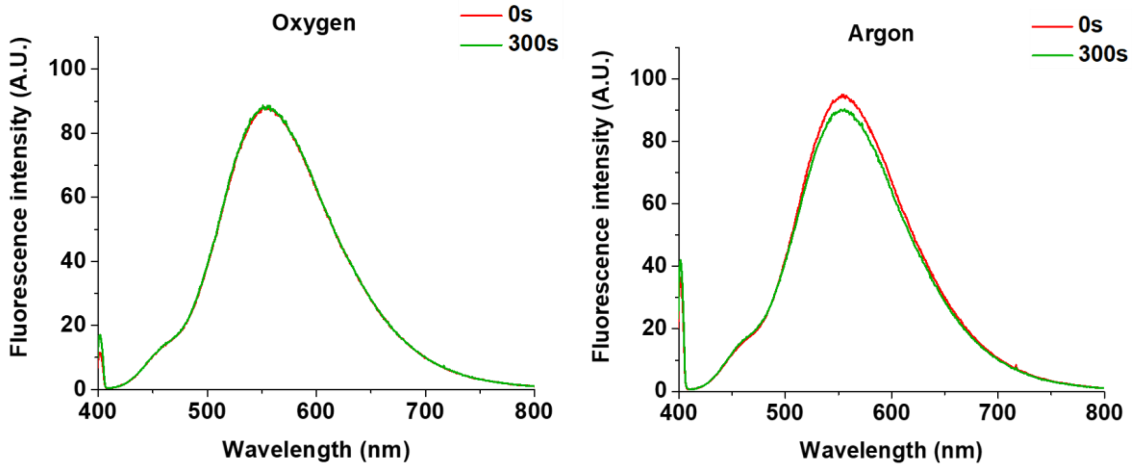

Figure S9. Emission spectra of **SC-P** in without any irradiation in the presence and absence of oxygen. Two methanolic solutions of **SC-P** (5 μM) were kept in the dark for 300s, one under air atmosphere and the other under argon atmosphere. The emission spectra were acquired right away and after 300 s.

5) $^1O_2$ **dependency**

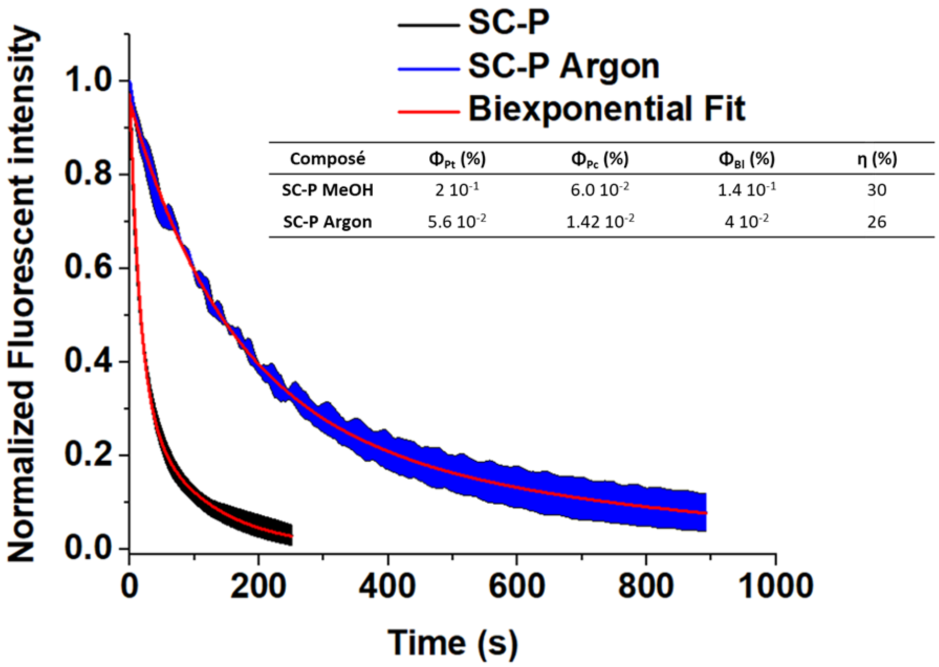

Composé	Φ_{Pt} (%)	Φ_{Pc} (%)	Φ_{Bl} (%)	η (%)
**SC-P MeOH**	$2\ 10^{-1}$	$6.0\ 10^{-2}$	$1.4\ 10^{-1}$	30
**SC-P Argon**	$5.6\ 10^{-2}$	$1.42\ 10^{-2}$	$4\ 10^{-2}$	26

Figure S10. Decay of fluorescence intensity of **SC-P** in methanol and in argon degassed methanol monitored over the time and upon 488 nm laser irradiation (33 mW/cm^{-2}).

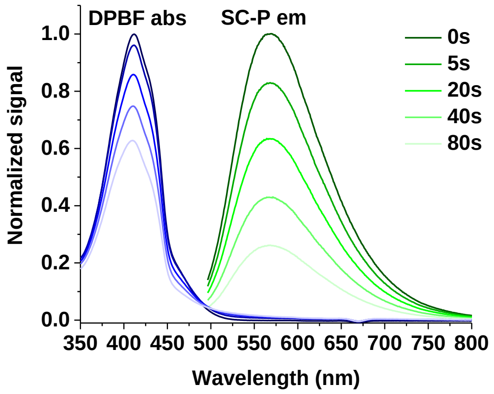

Figure S11. Normalized absorption and emission spectra of DPBF (singlet oxygen sensor) and **SC-P** upon excitation of **SC-P** showing that $^1O_2$ is generated upon irradiation. Singlet oxygen generation study was carried out by irradiated of solution containing 100 µM of DPBF and 5 µM of **SC-P** in MeOH with a 488 nm laser (33 mW/cm²). The absorption and emission spectra were recording over time.

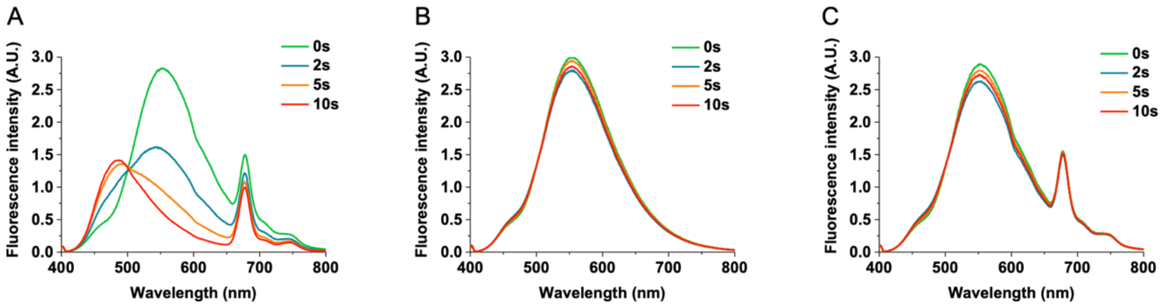

Figure S12. Irradiation experiments to show the reactivity of **SC-P** towards singlet oxygen. (A) Conversion of **SC-P** by undirect formation of singlet oxygen through excitation of Aluminum Phthalocyanine AlPh (excitation at 638 nm).[11] (B) irradiation without AlPh at 638 nm. Evolution of **SC-P** spectrum over the time in the presence of AlPh without excitation at 638 nm. The spectra were acquired under a 405 nm laser excitation.

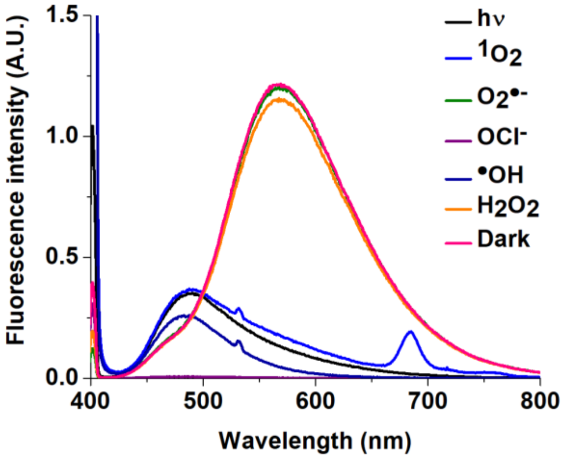

Figure S13. Emission spectra of **SC-P** in the presence of various ROS, without ROS in the dark (negative control) and without ROS after irradiation (positive control). Selectivity tests were performed adding various ROS generators in water in a methanolic solution of **SC-P** (5 µM). $^1O_2$: 5 µM of Aluminium Phthalocyanine with 638 nm laser for 10 s: $O_2^{\bullet -}$: 2 mM of KO_2 for 30 minutes in the dark; OCl^-: 2 mM of NaOCl for 30 minutes in the dark; $^{\bullet}OH$: 2 mM of $FeSO_4$ + 200 µM H_2O_2 for 30 minutes in the dark; H_2O_2: 2 mM of H_2O_2 for 30 minutes in the dark. The emission spectra were acquired with 405 nm laser.

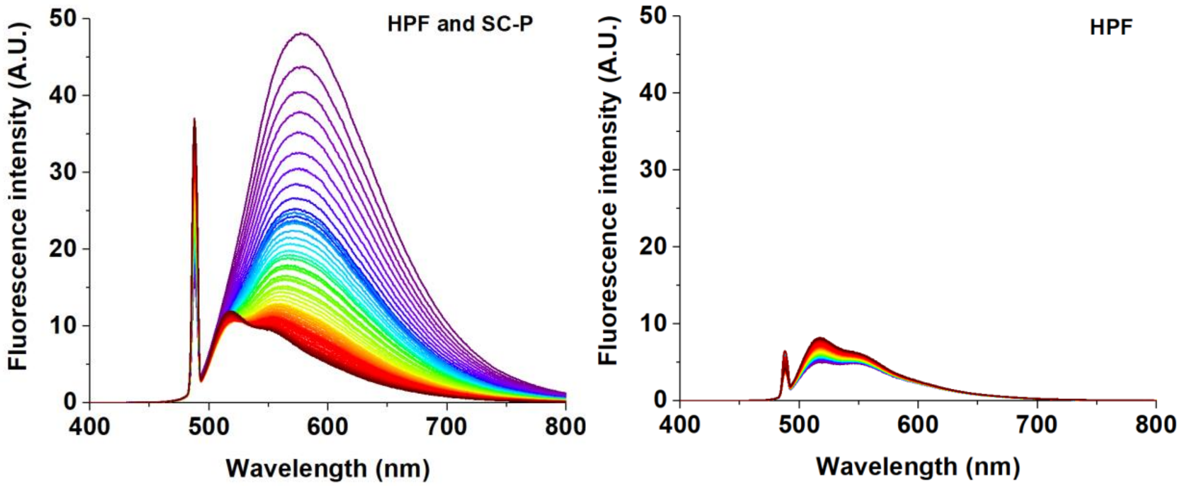

Figure S14. Emission spectra of HPF in presence (left) and in the absence of **SC-P** (right) under irradiation at 488 nm showing that •OH is not generated upon irradiation and conversion. Hydroxyl radicals generation study was carried out by irradiated of solution containing 20 µM of HPF and 5 µM of **SC-P** in 50/50 : MeOH/PBS with a 488nm laser (33 mW/cm²). The emission spectra were recording over time.

6) **Fluorescence decay curves**

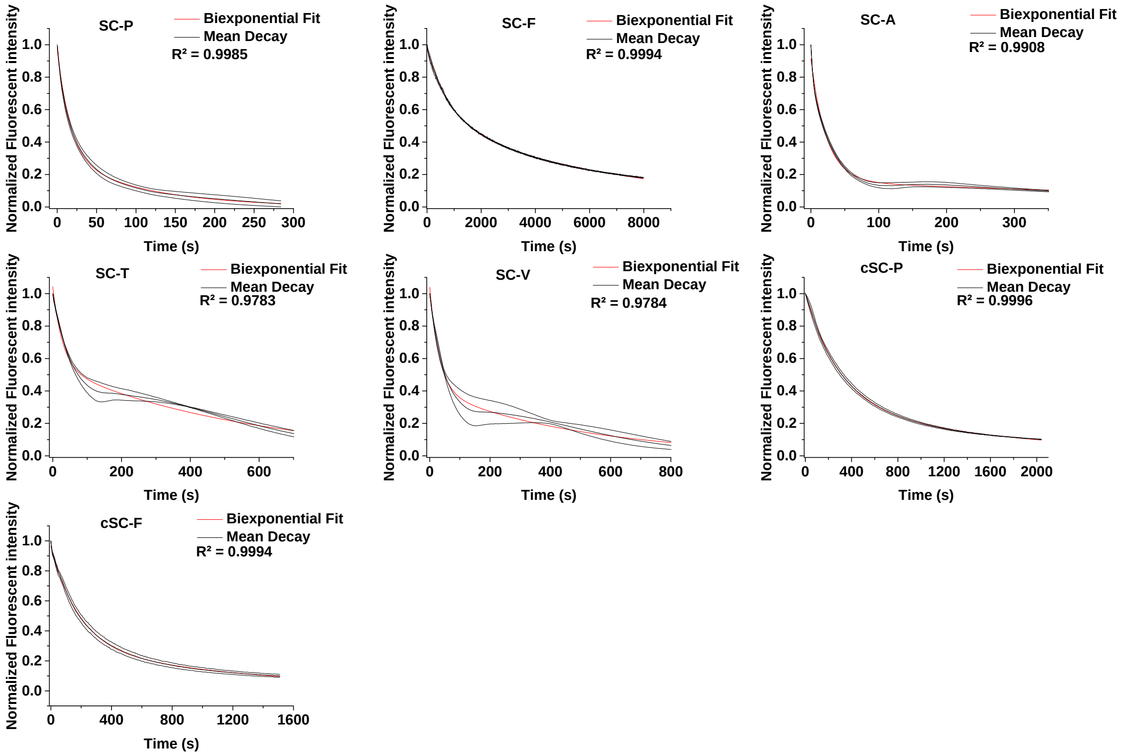

Figure S15. Decay of fluorescence intensity monitored over the time and upon laser irradiation of the **SCs** and their converted for **cSCs**. The grey areas represent the error bars obtained with triplicate experiences. The red curve is the biexponential fit. R^2 represents the goodness of the fit.

7) Cytotoxycity phototoxicity assays

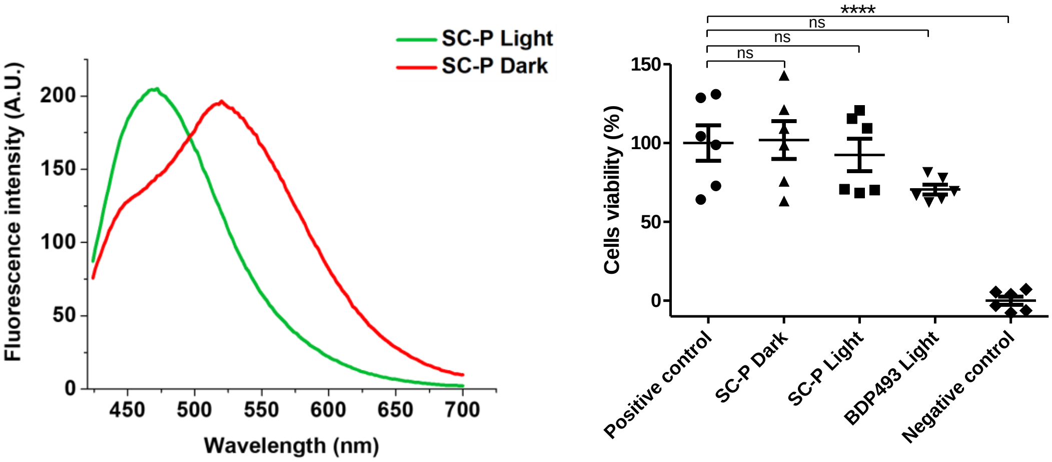

Figure S16. Emission spectra (380 nm excitation in plate reader) of plated cells incubated with **SC-P** before and after irradiation with 475 nm LED proving that the conversion occurs under these conditions. Cells viability under different conditions. For the 3-(4,5-Dimethylthiazol-2-yl)-2,5-Diphenyltetrazolium Bromide (MTT) assay, HeLa cells were incubated with **SC-P** (1 µM) in a 96 wells plate. The conversion was performed by irradiating each well using a 5× objective and a green LED (475/40 nm LED from X-cite 110 Led illumination system) for 1 min. The cells were then incubated at 37°C. After one hour the medium was removed and MTT (Sigma Aldrich) at 0.5 mg/mL in PBS was added to cells and incubated 3 hours. After the formation of the subtract to a chromogenic product by metabolically active cells, the medium was removed and 100 µL of DMSO was added to solubilized the crystals. The absorbance of each samples was measured with a plate reader (TECAN) spectrometer at 570 nm. Cell viability was reported as relative decrease compared to the absorbance of the positive control (Cells without **SC-P** and light) considered as 100% of viable cells and negative control (Cells with Tryton 0.1%) considered as 0% of viable cells. Data are presented as mean ± standard error of the mean of six independent experiments. As comparison with a non-photoconvertible probe, BDP 493, a common green emitting lipid droplet marker was used at 500 nM. ns: non-significative, ****$p<0.0001$

8) **Fluorescence microscopy studies**

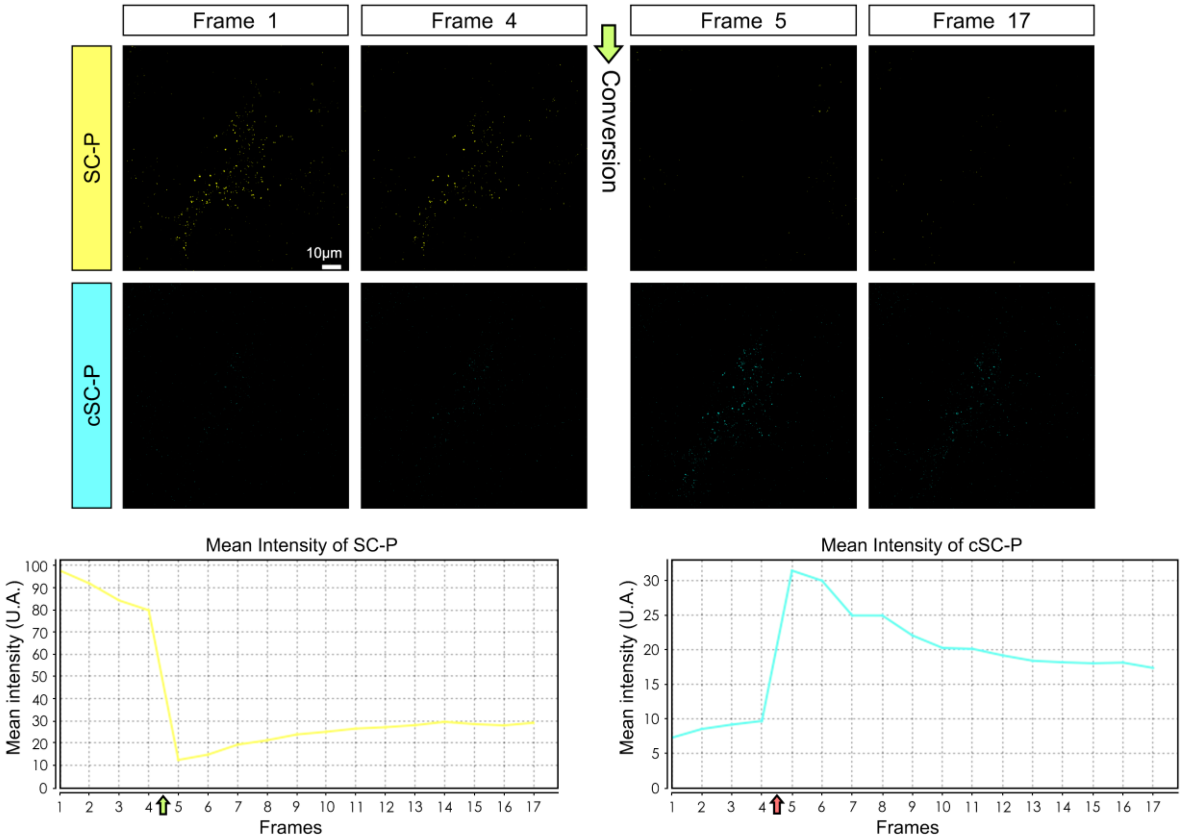

Figure S17. Conversion at a desired timepoint. Images of SC-P labeled LDs in HeLa cells before and after conversion (at frame 5) and the intensity profile of both SC-P and cSCP-P channels over 17 frames. SC-P labeled LDs can be imaged during several frames without undesired conversion allowing to trigger conversion at a desired timepoint.

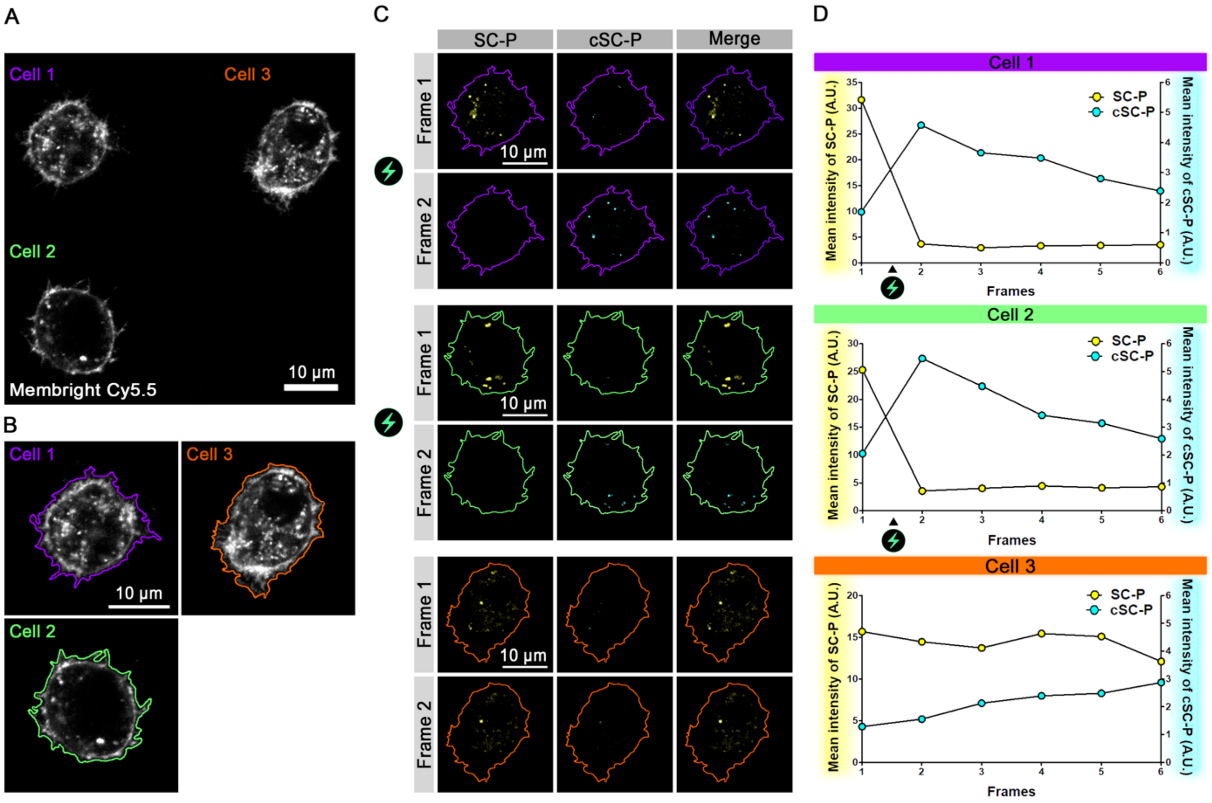

Figure S18. Selective conversion of SC-P in Hela cells. (A) Membright Cy5.5 labeling was used to track cell shape over time. (B) HeLa cells 1 and 2 were converted whereas cell 3 was not stimulated. (C) Results of photoconversion (green flash) is shown on frame 2 for each cell. **SC-P** form and converted **cSC-P** form are shown in yellow and cyan respectively. (D) Quantification of the mean intensity of initial **SC-P** form and converted **cSC-P** form after irradiation and over time.

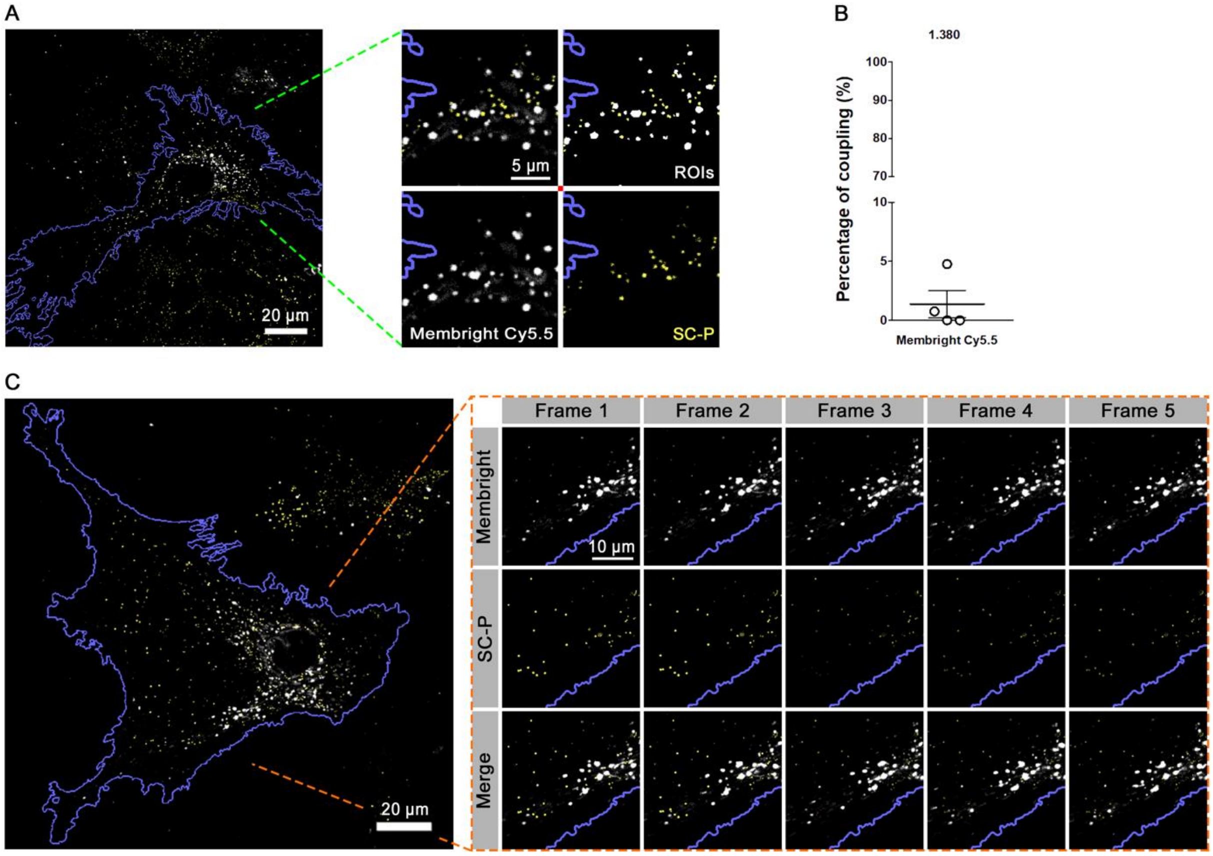

Figure S19. Tracking of SC-P with MemBright Cy5.5-labeled intracellular vesicles over time in astrocytes. (A) **SC-P** and MemBright Cy5.5 were incubated 2 h at 37°C, to monitor plasma membrane internalization. Endocytosed plasma membrane is visualized as white MemBright positive endosomes within the cells which are distinct from **SC-P** spots confirming that **SC-P** is specifically enriched in lipid droplets and not internalized in endosomes. (B) Colocalization analysis showed very few association (1,38%) of **SC-P** (yellow) with internalized MemBright probe (white) with a non-significant p value (>0,05) indicating a fortuitous association due to spot density. (C) Time lapse showing **SC-P** vesicles and MemBright vesicles within astrocytes along time, showing absence of colocalization even after several frames.

9) References

[1] D. Ray, P. K. Bharadwaj, *Inorg. Chem.* **2008**, *47*, 2252–2254.
[2] K. Renault, P.-Y. Renard, C. Sabot, *Eur. J. Org. Chem.* **2018**, *2018*, 6494–6498.
[3] R. Ambre, C.-Y. Yu, S. B. Mane, C.-F. Yao, C.-H. Hung, *Tetrahedron* **2011**, *67*, 4680–4688.
[4] B. M. Trost, C. A. Kalnmals, D. Ramakrishnan, M. C. Ryan, R. W. Smaha, S. Parkin, *Org. Lett.* **2020**, *22*, 2584–2589.
[5] J. Gordo, J. Avó, A. J. Parola, J. C. Lima, A. Pereira, P. S. Branco, *Org. Lett.* **2011**, *13*, 5112–5115.
[6] S. Padmanabhan, R. Peri, A. Rutledge, D. J. Triggle, *Journal of Heterocyclic Chemistry* **1997**, *34*, 301–304.
[7] A. Chatterjee, D. Seth, *Photochem Photobiol* **2013**, *89*, 280–293.
[8] M. A. Thompson, J. S. Biteen, S. J. Lord, N. R. Conley, W. E. Moerner, in *Methods in Enzymology*, Elsevier, **2010**, pp. 27–59.
[9] F. de Chaumont, S. Dallongeville, N. Chenouard, N. Hervé, S. Pop, T. Provoost, V. Meas-Yedid, P. Pankajakshan, T. Lecomte, Y. Le Montagner, T. Lagache, A. Dufour, J.-C. Olivo-Marin, *Nat Methods* **2012**, *9*, 690–696.

[10] T. Lagache, A. Grassart, S. Dallongeville, O. Faklaris, N. Sauvonnet, A. Dufour, L. Danglot, J.-C. Olivo-Marin, *Nat Commun* **2018**, *9*, 698.
[11] T. Nyokong, V. Ahsen, Eds. , *Photosensitizers in Medicine, Environment, and Security*, Springer Netherlands, Dordrecht, **2012**.
